# qPCR assay optimisation for a clinical study comparing oral health risk in Rett syndrome

**DOI:** 10.1007/s40368-024-00912-8

**Published:** 2024-06-26

**Authors:** Y. Y. L. Lai, J. Downs, S. Leishman, H. M. Leonard, L. J. Walsh, S. Zafar

**Affiliations:** 1 UQ Oral Health Centre, The University of Queensland School of Dentistry, 288 Herston Rd, Herston, QLD 4006 Australia; 2 Child Disability, Telethon Kids Institute, PO Box 855, West Perth, WA 6872 Australia; 3 Curtin School of Allied Health, Curtin University, GPO Box U1987, Perth, WA 6845 Australia

**Keywords:** *Streptococcus mutans*, *Scardovia wiggsiae*, *Porphorymonas gingivalis*, *Corynebacterium durum* and *Rothia dentocariosa/aeria*, *Fusobacterium nucleatum* subsp.* fusiforme*

## Abstract

**Purpose:**

This study aimed to validate qPCR assays for specific microbiota, for use on dental plaque samples stored on Whatman FTA cards to compare relative oral health risk in Rett syndrome.

**Methods:**

Supragingival dental plaque samples were collected, using a sterile swab, (COPAN FLOQswab™) swabbed onto Whatman FTA™ cards. DNA extraction was performed using a modified Powersoil™ protocol. Where published assays were unsuitable, species-specific qPCR assays for caries-associated, gingivitis-associated and oral-health-associated bacteria were designed using multiple sequence alignment, Primer3Plus and PrimerQuest. Assays were run using absolute quantification. Limit of detection (LOD) and limit of quantification (LOQ) were calculated, and PCR products verified by Sanger sequencing.

**Results:**

Most assays allowed detection using real-time qPCR with high specificity on samples collected on FTA cards. Several assays showed low or even single gene copy numbers on the test samples.

**Conclusion:**

Assays were optimised for detection and evaluation of oral health risk in dental plaque samples stored on FTA cards when cold storage is not feasible, except for *F. nucleatum*. Several assays showed gene copy numbers less than the LOQ or outside the range of the standard curve, so there is merit in optimising these assays using digital droplet PCR.

**Supplementary Information:**

The online version contains supplementary material available at 10.1007/s40368-024-00912-8.

## Introduction

Oral health risk assessment is an important tool to inform the dental management and the balance between preventive and aggressive management approaches. Currently, AAPD CRAF (American Academy of Pediatric Dentistry 2022) tool and ADA CRA tool (American Dental Association 2023a, b) both list special healthcare needs as a moderate or high-risk factor for dental caries. In general, while there are known medical conditions that we know modify risk factors, the current body of knowledge is not exhaustive. Specifically, there are a number of conditions, such as intellectual and developmental disabilities (IDD), where the oral health risk relative to the general population is not known. For example, there are (Dieguez-Perez et al. 2016) conflicting suggestions on the effect of salivary pH and salivary osmolality on dental caries risk (Ruiz et al. 2018; Santos et al. 2014). Similarly, other conflicting systematic reviews exist for autism spectrum disorder (ASD) (Da Silva et al. 2017; Lam et al. 2020).

Given the dearth of knowledge on the IDD conditions, it is with less certainty that one can accurately comment on how other rarer IDD conditions function as effect modifiers on oral health risk and how it influences that individual’s oral health trajectory. The use of an objective means to measure relative oral health risk would be useful to balance preventive with more aggressive oral health management approaches on a population-based level for a targeted disability sub-group where clinical examination may be difficult. One objective approach to assessing oral health risk has involved quantification of selected bacteria in the oral microbiome as representative oral health indicators, through applications, such as quantitative real-time polymerase chain reaction (RT-qPCR), where published assays have been designed either for primer-based or probe-and-primer-based qPCR reactions. After all, of the validated CRATs that exist, only CAMBRA includes bacterial culture counts as a modifying risk factor, and for *mutans streptococci* and *lactobacillus spp.* only. For dental caries, one commonly used bacteria is *Streptococcus mutans*, which is a known bacterial species present in many cases of dental caries. More recently, *Scardovia wiggsiae* has been identified as a bacterial species present in dental caries even in the absence of *S. mutans* (Isaac et al. 2022), and *Porphyromonas gingivalis* has been identified as being present in gingivitis. One known indicator of dental health is *Corynebacterium durum* (Dinakaran et al. 2018), as well as *Rothia dentocariosa*, a low-virulence commensal generally considered an indicator for both dental and gingival health (Yeung et al. 2014) but found in greater amounts in those with high caries risk (AlEraky et al. 2021). Another indicator of gingival health is the strain *Fusobacterium nucleatum subsp. fusiforme*, or more recently re categorised as *F. nucleatum subsp. vincentii* (Gharbia et al. 1990; Lourenço et al. 2014).

One advantage of quantifying and comparing oral bacterial species to assess oral health risk is that it provides a quantitative approach and can therefore be useful to evaluate comparative oral health for rare diseases. However, this would require collection of dental plaque samples and accepted protocols usually utilise cold storage at the time of collection. This is a barrier for sample collection in rarer diseases due to low birth prevalence and potential requirement for extensive travel for sample collection, particularly in countries with sparser population distribution. Currently, room temperature storage of samples for up to 17 years is possible using archival cards (Whatman FTA cards), which contain chemical reagents to lyse bacteria and denature proteins, and rapidly inactivate microorganisms, preventing the growth of bacteria, whilst also protecting nucleic acids from nucleases, oxidation and UV damage. Using these cards allows capture of the nucleic acids required for later molecular analysis in one step, and they remain immobilised in the card and preserved for both transport and long-term storage at room temperature (Aldrich 2020). However, storage of dental bacterial samples on FTA cards, and subsequent DNA extraction for use in qPCR, has not been reported and validated in the literature. Furthermore, existing protocols for swabbing times prior to FTA card storage for those with IDD may be challenging due to compliance. The low DNA yields expected from samples of individuals with special healthcare needs, and potentially low DNA yields from other DNA extraction methods for bacterial samples, would necessitate testing of samples collected using tried methods such as nylon flocked swabs, as well as samples containing known or high quantities of bacteria as positive controls as proof of concept of the efficacy of DNA extraction from FTA cards which have not previously been used for this application or published elsewhere in the literature.

Another challenge with using qPCR assays to evaluate oral bacterial species is that the specificity of the approach is limited by the specificity of the *in-vivo* assay design, which must meet strict parameters before wet-lab testing, adding to the expense, particularly for assays using a hydrolysis probe. Further, an extensive literature search was conducted for existing assays that were either species or strain-specific. This revealed that many published sequences were problematic. For several publications, the amplicon target was not found in the corresponding accession number or *FASTA* sequence (Avila‐Campos et al. 1999; Lochman et al. 2019), even when the version number was given. In other publications, the left or right primer was missing entirely from the target amplicon (Sakamoto et al. 2001; Topcuoglu et al. 2013; Tran and Rudney 1999; Yoshida et al. 2003), or at least one of the primers had a high mispriming score (Ammann et al. 2013; Kim et al. 2004; Park et al. 2013) or had an unacceptable melting temperature (Tm) (Tanner et al. 2011). Importantly, many primer sets had multiple off-targets when checking with *primer-BLAST*. Most assays used only primers and not probes.

The two major problems, being a lack of specific qPCR assays and lack of a tried and tested approach for prolonged dental sample storage at room temperature for qPCR testing, were the barriers the authors wished to overcome. This prompted us to undertake a proof-of-concept study involving interstate travel across Australia to collect and compare dental plaque samples from both individuals with Rett syndrome (RTT), and their sibling control participants, to gain more accurate assessment of their relative oral health risk where there is a lack of information. Rett syndrome is an X-linked neurodevelopmental disorder mainly affecting females (Neul et al. 2010) that occurs in approximately 1 in 9000 female live births (Fehr et al. 2011). *MECP2* is the main gene implicated (Amir et al. 1999). Patients with RTT develop progressive cognitive impairment after a period of relative normalcy, and experience severe or profound intellectual disability. In a recent literature review, the research team has summarised the current state of knowledge with respect to oral health problems in RTT (Lai et al. 2018a, b). In a recent study, our team examined the oral health experiences of patients with RTT by accessing data on the Australian Rett Syndrome Database (ARSD) (Lai et al. 2018b). A major deficit that we identified in the literature is a lack of comparisons to healthy controls. Only one small clinical study to date included a control group (Fuertes-Gonzalez and Silvestre 2014), involving clinical examination but no microbial analyses of oral microflora.

Therefore, with this in mind, this study aimed to develop a suite of qPCR assays for specific microbiota as indicators of oral health and disease (namely, *S. mutans, S. wiggsiae, C. durum, R. dentocariosa, P. gingivalis*, and *F. nucleatum* subsp*. fusiforme;* and to test the suitability of FTA cards for oral bacterial sample storage and test DNA extraction of oral bacterial samples from FTA cards for use in real-time qPCR as a proof-of-concept for application in a future study to compare oral microflora collected from samples collected from those with RTT and their sibling controls.

It was hypothesised that DNA extraction of bacterial DNA from dental plaque samples stored on Whatman FTA cards would be comparable to DNA extracted of bacterial DNA from dental plaque samples stored on the nylon swabs used to transfer the sample from the mouth to the FTA card, and that the DNA extracted from bacterial DNA from dental plaque samples stored on Whatman FTA cards would be sufficient quantity and quality for downstream analysis using real-time qPCR. It was also hypothesised that real-time qPCR assays designed for *S. mutans, S. wiggsiae, C. durum, R. dentocariosa, P. gingivalis*, and *F. nucleatum* subsp*. fusiforme* would be specific, and would run with an efficiency between 90 and 110%, using DNA extracted from Whatman FTA cards.

## Materials and methods

### Research project setting

Ethics approval was received from The University of Queensland Human Research Ethics Committee (Approval No. HE2019002816).

This study focused on the optimisation and validation process for six qPCR assays using samples collected as per the protocol used for a multi-centre study comparing oral health of those with RTT with sibling controls. The multicentre study protocol is summarised herein to provide context to the downstream application of this proof-of-concept validation study. Briefly, the multi-centre study compared oral health status and oral health risk in individuals with RTT and sibling controls. Because of the location of treatment centres for RTT, this part of the study was multi-centre. The same clinician (YL) conducted dental examinations of individuals with RTT and siblings as controls at participants’ homes across the different sites. This part of the project included assessments of plaque factors that mediate the risk of oral diseases, the focus of *this* validation study.

#### Multicentre study sample population

This consisted of those with RTT residing in Western Australia, South Australia and Queensland, registered with the population-based Australian Rett Syndrome Database (ARSD). Home visits occurred in January–February 2021 and November 2022 (South Australia), May 2022 (Western Australia) and June 2022 (Queensland). For the control population, medically healthy siblings who were living in the same household, or nearby suburb (if no siblings), without disability were recruited concurrently. Target recruitment numbers were based on approximately half the number of families residing within 1–1.5 h’ drive from the reference city centre, as indicated in Supplement 1 Table 1, with the total number of those with RTT (alive) and living in this area provided as reference. Considering geographical factors and the rarity of the disease, the conservative recruitment targets were considered reasonable. A short questionnaire was disseminated to inform a clinical examination. This was adapted from a previous version of a dental module of the *InterRett* database (Lai et al. 2022, 2021, 2023). Participants were contacted via telephone to discuss the study and any questions that arose before providing written consent.

**Table 1:** qPCR assay oligonucleotide and target information

Assay set	Publication	Accession	Specificity	Target gene	Type		Start	bp	Tm	GC %	Purification
*S. mutans* UA159 gtfB	This study	UA159	Species specific	gtfB	Forward Primer	TGACTTGCTCCAAATTGCTG	1374	20	60	45	Standard desalting
	This study				Probe	/5SUN/GC ATG GAG T/ZEN/G ACA ACG ACA C/3IABkFQ/	1468	20	60.2	55	HPLC
	This study				Reverse Primer	TGCCTGAACGTTGATTTAAGG	1591	21	60.1	42.9	Standard desalting
					Product		1374	218			
*S. wiggsiae* spp. 16 s	This study	URS00004C5BB1 (RNA central)	Species specific	16S	Forward Primer	TGGTGAGTGGACTTTATGAATAAGCACCGG	442	30	71.5	46.7	Standard desalting
	This study				Probe	/56-FAM/AT TTA TTG G/ZEN/G CGT AAA GGG C/3IABkFQ/	524	20	60.2	45	HPLC
	This study				Reverse Primer	CAGCGCAACCTACCGTTAAGCAGTAAGCTT	62	30	71.5	50	Standard desalting
					Product		442	161			
*P. gingivalis* spp. hmuY	Gmiterek et al. 2013		Species specific	hmuY	Forward Primer	GCTTCGAAATACGAAACGTG	133	20	58.4	45	Standard desalting
	This study				Probe	/56-FAM/CG CTA TGA C/ZEN/G TTC GTC TCA A/3IABkFQ/	235	20	60	50	HPLC
	Gmiterek et al. 2013				Reverse Primer	TATATCCGTCTGTCGGAACG	346	20	58.6	50	Standard desalting
					Product		133	214			
*C. durum* spp. 16 s	This study	Z97069	Species specific	16S	Forward Primer	GGCTGTGTGTTTGCAGTCTG	720	20	60.5	55	Standard desalting
	This study				Probe	/5SUN/GC GGA GCA T/ZEN/G TGG ATT AAT T/3IABkFQ/	828	20	59.9	45	HPLC
	This study				Reverse Primer	TAAGGTTCTTCGCGTTGCAT	869	20	60.8	45	Standard desalting
	This study				Product		720	150			
*F. nucleatum* subsp. *fusiforme* 16 s	Shin et al 2010	AY185204	Strain specific	16S	Forward Primer	GATGAGGATGAAAAGAAACAAAGTA	5124	25	58	32	Standard desalting
	This study				Probe	/56-FAM/TG AAT TAA A/ZEN/G AAA GAA GTG GCA A/3IABkFQ/	5282	23	58.1	30.4	HPLC
	Shin et al 2010				Reverse Primer	CCATTGAGAAGGGCTATTGAC	5516	21	58.7	47.6	Standard desalting
					Product		5124	393			
*Rothia dentocariosa*/*Rothia aeria*	This study	GU416730.1	Species specific	16S	Forward Primer	CGTGAGTGACCTACCTTTGAC	39	21	62	52.4	Standard desalting
	This study				Probe	/56-FAM/CT GGG ATA A/ZEN/G CCT GGG AAA CTG GG/3IABkFQ/	61	24	68	58	HPLC
	This study				Reverse Primer	CATAACGCTTTCCACCAACAC	118	21	62	47.6	Standard desalting
	This study				Product		39	100			

### Data collection

All participants’ families were contacted prior to each home visit. Selected additional participants also provided consent for taking of samples which were used for the assay optimisation stage which is the focus of this study.

**Fig. 1:**
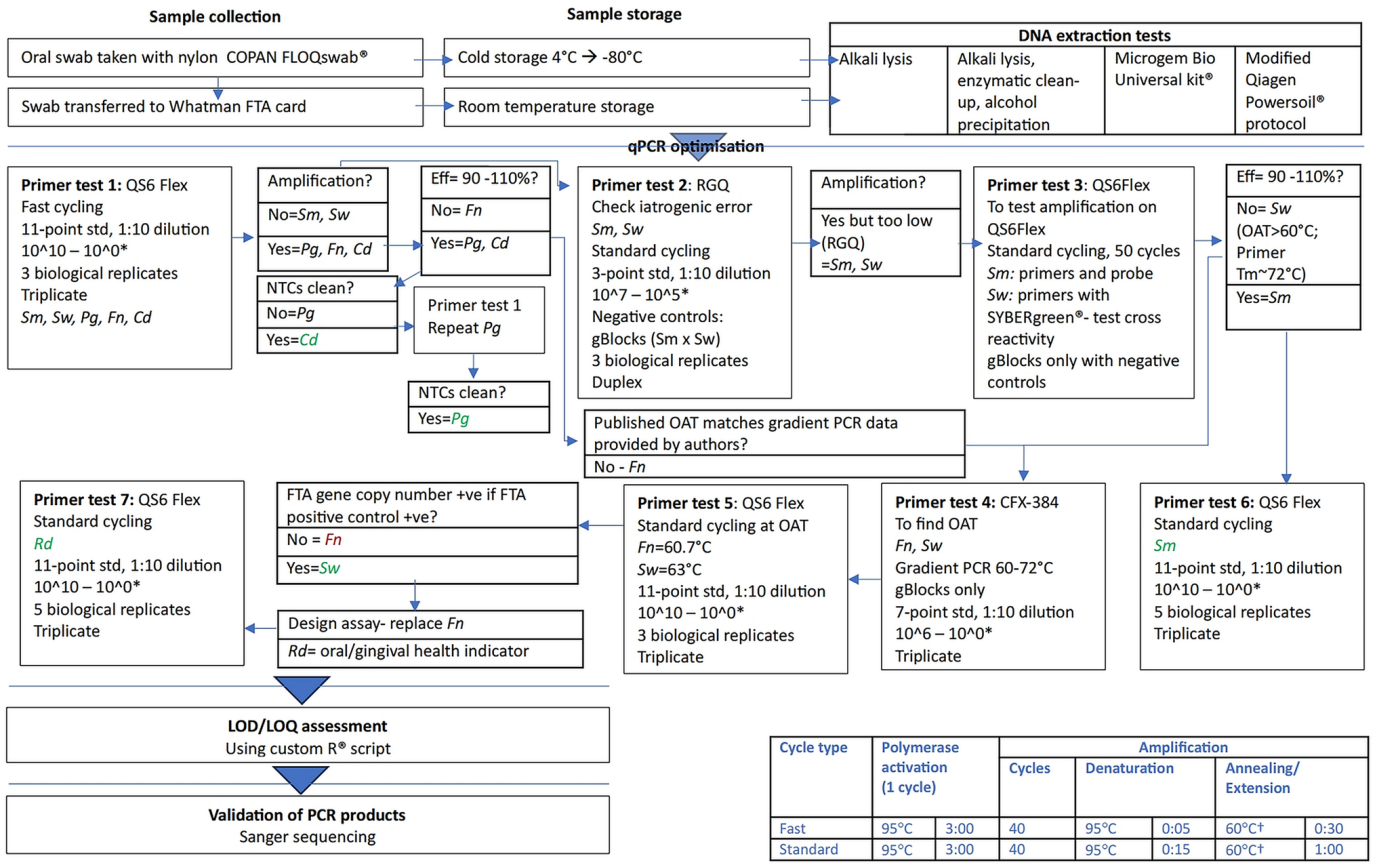
Flow diagram illustrating workflow of proof-of-concept study. Legend: † Unless otherwise specified Cd = C. durum; CFX-384 = CFX-384 (Biorad): analysed with Bio-Rad CFX Maestro 1.0 Version 4.0.2325.0418 (Bio-Rad). Eff = qPCR efficiency; Fn = *F. nucleatum* subsp. fusiforme; LOD/LOQ = limit of detection/limit of quantification; *NTC* no template control; *OAT* optimal annealing temperature; *PCR* polymerase chain reaction; *Pg* = *P. gingivalis*; QS6 Flex Quantstudio 6 Flex; *RGQ* RotorGene Q 5-Plex HRM (Qiagen), analysis with Rotor-Gene Q Series Software v2.3.5 (Build 1) (Qiagen) with manual pipetting (not compatible with robotic pipetting).; Rd = *Rothia dentocariosa*/*R. aeria*; Sm = *S. mutans*; Std = standard; Sw = *S. wiggsiae*; *Shorthand notation for gene copy dilutions are based on the following calculated gene copies for bacterial targets at 10^10 gene copies: Cd = 12,833,183,536.50; Fn = 11,261,404,605.00; Pg = 19,198,587,102.00; Rd = 17,428,077,501.00; Sm = 18,903,502,168.50; Sw = 12,128,592,981.00

The methods described below related directly to this optimisation and validation study. The clinical workflow is illustrated in Fig. 1.

Dental plaque was collected for the assessment of microbiome dysbiosis that relates to the risk of dental caries (Philip et al. 2018). Briefly, supragingival dental plaque was collected with a sterile swab of the labial surface of the upper central teeth (or adjacent teeth if these were missing) and swabbed onto indicating archival storage cards (Micro FTA Indicating Cards, Whatman, Cat. No. WHATWB120211). The sterile swabs were placed in cold storage immediately, and transferred to − 20 °C storage within 4 h of data collection. Samples were later transferred to − 80 °C storage when practicable. The FTA cards were stored at room temperature, in line with manufacturer’s instructions.

### Optimisation of DNA extraction process

Selected samples were used to check both the quality and quantity of DNA from DNA extraction. Several DNA extraction protocols were tested, as described below.

#### Method 1. Modified Whatman extraction method b protocol

A Qiagen Harris Unicore 2 mm micro-punch was used to isolate bacterial DNA for use in downstream extraction from classic FTA cards. Extractions were performed according to the Whatman protocol for alkali lysis from classic FTA cards Method B (Miles and Saul 2015) with modifications to the sample size and reagent volumes. The entire 30 mm diameter FTA samples were cut into small sections approximating 1 × 1 mm with a sterile surgical blade or surgical scissors that had been disinfected with 70% ethanol and DNAOUT™ DNA Removal Solution. The samples were washed with 600 µL of Qiagen FTA Wash solution (Cat # 2,719,978) and incubated for 5 min at room temperature (RT) with occasional mixing with an Eppendorf Thermostat C heating and cooling block heater. The supernatant was removed and washing procedure was repeated a further three times. The samples were then washed three times with 600 µL of TE buffer (10 mM Tris pH 8.0, 1 mM EDTA), and the tubes were incubated for a further 5 min at RT with occasional mixing and the liquid was removed with a pipette. The buffer was removed and 35 µL of alkaline incubation buffer added (0.1 N NaOH, 0.3 mM EDTA, pH 13.0). Samples were incubated at 65 °C (Method B) for 5 min. Then, 65 μL of neutralising solution (0.1 M Tris–HCl, pH 7.0) was added and the samples vortexed 5 times. Tubes were incubated for 10 min at RT. The tubes were then mixed by pulsed vortexing 10 times. The FTA samples were removed from the solution, ensuring that the maximum amount of elute was recovered.

#### Method 2. DNA extraction followed by enzymatic cleanup and ethanol precipitation

DNA extraction was performed using the modified Whatman Extraction Method B outlined above, with additional repurification steps using Proteinase K and RNase A, followed by an ethanol precipitation step. Steps 2 and 3 of the New England Biolabs (NEB) 'Enzymatic Cleanup Protocol (removal of proteins and/or RNA) (New England Biolabs) were followed by an ethanol precipitation step to remove protein, RNA and residual chemical contaminants. Briefly, 1μL of Proteinase K and 1μL RNase A was added. Samples were mixed briefly and incubated at 56 °C for 5 min. For DNA ethanol precipitation, 0.1 vols 3 M sodium acetate was added to 2.5–3 vols ice cold 100% ethanol, and vortexed to mix thoroughly. Samples were precipitated at -20 °C overnight and centrifuged at full speed (14,000 rcf), 4 °C for 20 min. The pellet was washed twice with 0.5 mL ice cold 70% ethanol and centrifuged at 14,000 rcf at 4 °C for 10 min each time. The ethanol was removed by centrifuging quickly (10 s at 14,000 rcf). The pellet was air dried for 10 min and resuspended in 20 μL of nuclease-free water (1.000 g/mL at 3.98 °C (litre)). Finally, the pellet was incubated at 65 °C for 10 min with an Eppendorf Thermostat C heating and cooling block heater.

#### Method 3. DNA extraction using microgem bio Universal kit

DNA extraction was performed at the Australian Genome Research Facility Brisbane laboratory, on one test sample (“YL FTA”) using the manufacturer protocol (Miles and Saul 2015). Briefly, 100 µL of water was added to 2 mm diameter punches. Samples were briefly vortexed and placed at room temperature for 15 min. The tubes were then vortexed and the liquid removed by pipette. Punches were resuspended in: 44 µL nuclease-free water, 5 µL MicroGEM BLUE Buffer and 1 µL prepGEM. Tubes were then placed in a thermal cycler and heated at 75 °C for 15 min followed by 95 °C for 5 min. Finally, liquid was decanted into tubes.

#### Method 4. DNA extraction using a modified Qiagen Powersoil Kit protocol

DNA extraction was performed on a COPAN FLOQswab and its corresponding FTA card (approximately 2 mm punch), using the DNEasy Powersoil Pro kit (Qiagen) on an FTA sample and the corresponding swab sample.

DNA extraction was performed using a modified Powersoil protocol on samples labelled A19, B19 and C19, with the entire FTA sample included in the Powersoil reaction.

Preparation of the *Streptococcus sanguinis* culture was prepared as follows: 100 mL of Brain–Heart infusion (BHI) broth was prepared (3.7 g in 100 mL nuclease free water) and autoclaved at 121 °C. Then, 2 μL of *S. sanguinis* (ATCC 10556) culture was pipetted into 1 mL BHI broth and incubated for 48 h at 37 °C at 260 rcf on an Eppendorf Thermostat C heating and cooling block heater. The sample was centrifuged at 3900 rcf at 22 °C for 20 min to pellet the solution before discarding the supernatant. The pellet was resuspended in 100 μL nuclease-free water (1.0 g/mL at 3.98 °C (litre)). Then, half (50 μL) was pipetted onto an FTA card with sample label “B19” and allowed to sit for one hour. Samples B19 and C19 were then cut into small pieces approximating 1 × 1 mm with surgical scissors decontaminated with DNA-Out (Astral Scientific) followed by 70% ethanol, before including the entire samples in the Powersoil reaction. The other 50 μL of the *S. sanguinis* culture was then labelled as sample “A19”, and included directly into a Powersoil reaction.

### In silico design of qPCR assays

The study was conducted in line with the Minimum Information for Publication of Quantitative Real-Time PCR Experiments (MIQE) guidelines (Bustin et al. 2009). Custom qPCR assays (primers and probes) targeting two caries-associated bacterial species (*S. mutans, S. wiggsiae*), one gingivitis-associated bacterial species (*P. gingivalis*) and 3 health-associated commensal bacterial species (*F. nucleatum* subsp. *fusiforme, R. dentocariosa* and *C. durum*) were designed using Primer3Plus and PrimerQuest (IDT). Specificity of the assays and off-targets were assessed using Nucleotide BLAST and Primer BLAST, while the IDT Oligoanalyzer Tool was used for assessing melting temperature and primer dimer profiles.

The in silico design process involved a comprehensive literature search for published species-specific assays where possible, with target amplicons verified by multiple sequence alignment using Multiple Alignment using Fast Fourier Transform (MAFFT) software; or else assays were custom designed using multiple sequence alignment using MAFFT software to identify species-specific sequences, and these formed the basis for synthetic DNA templates (gBlocks standards) design. Primers, probes, and synthetic DNA templates (gBlocks standards) were custom manufactured by Integrated DNA Technologies (IDT).

The qPCR oligonucleotide and target information are detailed in Table 1. The qPCR information on gBlocks is detailed in Table 2.

**Table 2:** qPCR assay gBlocks sequences

Assay	gBlocks sequence
*S. mutans* UA159 gtfB	ACC GAG TTG CCC GTT AAA GTT GAC TTG CTC CAA ATT GCT GGG GAT TAC CTC AAA GCT GCT AAG GGG ATC CAT AAA AAT GAT AAG GCT GCT AAT GAT CAT TTG TCT ATT TTA GAG GCA TGG AGT GAC AAC GAC ACT CCT TAC CTT CAT GAT GAT GGC GAC AAT ATG ATT AAT ATG GAC AAT AAG CTG CGT TTG TCT CTA TTA TTT TCA TTA GCT AAA CCC TTA AAT CAA CGT TCA GGC AAG ATC CGA TGC GTA ACC GTT
*S. wiggsiae* spp. 16 s	AGA TCC GAT GCG TAA CCG TTT GGT GAG TGG ACT TTA TGA ATA AGC ACC GGC TAA CTA CGT GCC AGC AGC CGC GGT AAT ACG TAG GGT GCA AGC GTT GTC CGG ATT TAT TGG GCG TAA AGG GCT CGT AGG CGG TTT GTT GCG TCT GGT GTG AAA GCT TAC TGC TTA ACG GTA GGT TGC GCT GAA TGG CGT CCA GTT GTC ACA
*P. gingivalis* spp. hmuY	AAT GGC GTC CAG TTG TCA CAG CTT CGA AAT ACG AAA CGT GGC AGT ATT TCT CTT TTT CCA AAG GTG AAG TCG TAA ATG TTA CCG ACT ATA AGA ACG ATT TGA ACT GGG ACA TGG CTC TTC ACC GCT ATG ACG TTC GTC TCA ATT GTG GCG AAA GTG GCA AGG GAA AAG GTG GTG CCG TAT TCT CCG GCA AGA CAG AAA TGG ATC AGG CTA CTT CCG TTC CGA CAG ACG GAT ATA ACC AAT GGC TTT CCG AGA TG
*C. durum* spp. 16 s	ACC AAT GGC TTT CCG AGA TGG GCT GTG TGT TTG CAG TCT GTG CCG TAG CTA ACG CAT TAA GCG CCC CGC CTG GGG AGT ACG GCC GCA AGG CTA AAA CTC AAA GGA ATT GAC GGG GGC CCG CAC AAG CGG CGG AGC ATG TGG ATT AAT TCG ATG CAA CGC GAA GAA CCT TAA CCG AGT TGC CCG TTA AAG T
*F. nucleatum* subsp. *fusiforme* 16 s	ACC GAG TTG CCC GTT AAA GTG ATG AGG ATG AAA AGA AAC AAA GTA TAG AGA ACT CAA AAA CAA TTT TAA ATA ATA GAG ATA TAG AAA ATT TTA TAT CTA AAA ATA TGG CAA GTT ATA ACT TTA AGA AAA AAG AAG TTA TAT CAG AAT ATA AAC ATT CTT CAG TAG AAT GCT TCT CTT ATG AAT TAA AGA AAG AAG TGG CAA TTG ATA TAG ACA AAG AAA AAT TAT CTT TAA TAG TAA AAA ATA AAA TGA GAG ATT TGT ATA TAA AAG CTA ATT ATT TTG TAG ATG ATT TGT CAA AGG TTA TAG CTA AAG AAA AAC AGT ATA GAT TCT CTG ATA ATG ATG TAT CAG AAA ATC TTA AAG ACT ATG AAT ATT CAG ATA TAA AGA ATA TAG TAA AAG TCA ATA GCC CTT CTC AAT GGA CCA ATG GCT TTC CGA GAT G
*Rothia dentocariosa*/*Rothia aeria*	ACC GAG TTG CCC GTT AAA GTC GTG AGT GAC CTA CCT TTG ACT CTG GGA TAA GCC TGG GAA ACT GGG TCT AAT ACC GGA TAC GAC CAA TCT CCG CAT GGG GTG TTG GTG GAA AGC GTT ATG AGA TCC GAT GCG TAA CCG TT

### qPCR optimisation

Lysophilised samples were diluted according to manufacturer instructions. The qPCR protocol is summarised in Fig. 1. Primer (500 nM) and probe (250 nM) concentrations used the standard primer and probe concentrations recommended by the manufacturer (IDT), with a volume of 0.5 μL respectively. Primer and probe Mg2 + , and dNTP concentrations were 3 mM and 0.8 nM respectively. IDT PrimeTime Gene Expression Mastermix (IDT) was used with a volume of 5 μL, with 2 μL DNA template and 1.5 μL nuclease free water for a 10 μL reaction volume. Reaction setups were performed using an Assist Plus pipetting robot (Integra). An Applied Biosystems MicroAmp Optical 384-well reaction plate was used, in a Quantstudio 6 Flex (Applied Biosystems).

The DNA sample was mixed with an IDT Gene Expression (IDT) master mix, and robotically dispensed into a 384-well plate (10 μL/well, 2 µL DNA template/well). Synthetic DNA templates (gBlocks standards) served as the positive PCR control, and a non-template control was used to account for assay background. The gBlocks standards were run using a 1:10 dilution series from 10^0 to 10^10 dilutions on an 11-point standard curve, with the gBlocks concentrations shown in Fig. 1. Using test samples, optimised reactions were performed with the 384 well plate Quantstudio 6 Flex (Applied Biosystems). Assays were first run under the fast cycling conditions recommended by the manufacturer, and where assay efficiency was outside the range of 90–110%, assays were run under standard cycling conditions and gradient PCR was performed. Optimised assays were run with fast cycling conditions for *C. durum* and *P. gingivalis* assays, and on standard cycling conditions for *S. mutans*, and *R. dentocariosa/R. aeria*. The fast-cycling conditions were: enzymatic activation at 95 °C for 3 min, followed by 40 cycles of 95 °C for 5 s, and 60 °C for 30 s. The standard-cycling conditions were enzymatic activation at 95 °C for 3 min, followed by 45 cycles of 95 °C for 15 s, and 60 °C for 1 min. Samples of *S. wiggsiae* and *F. nucleatum* subsp. *fusiforme* were run at standard cycling conditions, but with optimal annealing temperatures of 63 °C and 60.7 °C respectively, which were determined by running a gradient PCR using CFX-384 qPCR instrument (Biorad).

### Data analysis

Data were analysed using Quantstudio software v1.2 (Applied Biosystems). The gene copy number in each sample was determined by comparing the CT values of each sample to those of the standards. Calibration curves for the primer tests were examined together with Cq (CT) variation, linear dynamic range, R2, and efficiency. The limit of detection (LOD) and limit of quantification (LOQ) were calculated using custom R scripts (Klymus et al. 2020). All assays were run in single plex.

### Verification of qPCR products

PCR products for the assays were confirmed by Sanger sequencing using broth cultures containing the target species *S. mutans* (ATCC25175), *F. nucleatum (subsp.* 49,256), *P. gingivalis* (W50)) and test samples where they could not be sourced as a broth culture (*S. wiggsiae*, *R. dentocariosa, C. durum)*.

The clinical workflow is illustrated in Fig. 1. For clarification for Primer Test 3, reactions were run in duplicate in 28 wells of 10 μL with setup as shown in Supplement 2 Table 1.

## Results

### DNA extraction results

The results for DNA extraction workflow are shown in Supplement 2 Table 2. These results showed that the modified Powersoil protocol was best compared to all other extraction methods in both DNA yield and quality.

### qPCR optimisation results

**Fig. 2:**
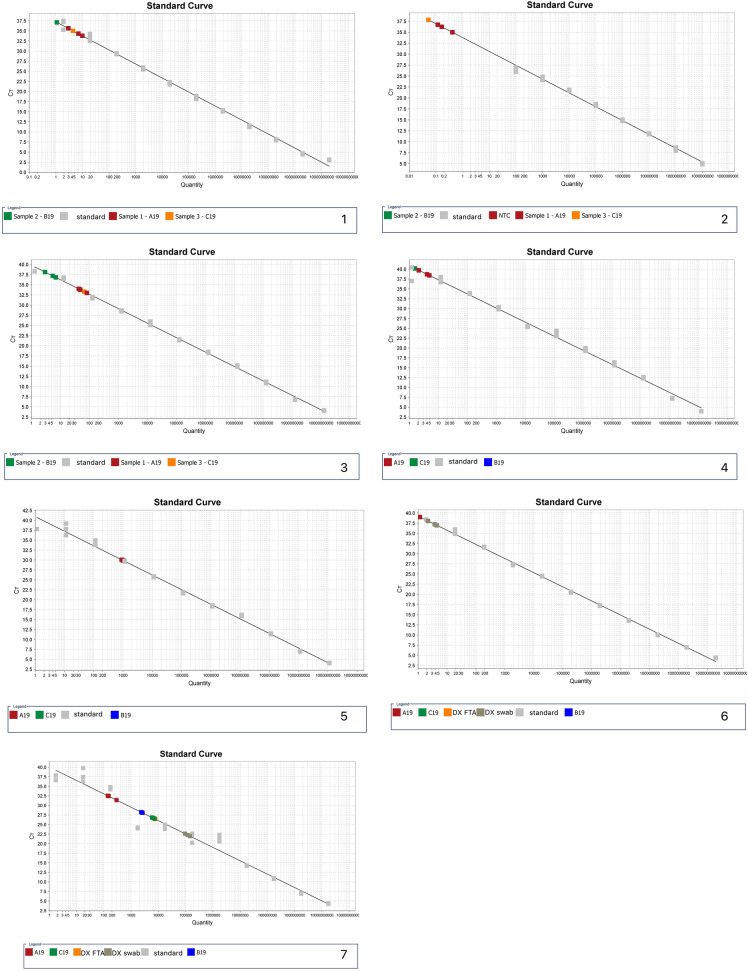
qPCR primer test amplification plots and standard curves. Unless otherwise stated, A = No Template Control; B through L are gBlocks standards from 10^10 through 10^0 respectively; M, N O and P (where applicable) are biological replicates. All reactions performed in triplicate. (1) *P. gingivalis* standard curve (2) Standard curve from rerun of *P*. *gingivalis* assay with 10^0, 10^1, and 10^10 dilutions omitted (3) *C. durum* standard curve (4) Standard curve for *S. wiggsiae* at optimal annealing temperature of 63 °C (5) Standard curve for *F. nucleatum* subsp. *fusiforme* at optimal annealing temperature of 60.7 °C (6) Standard curve for *S. mutans* (7) Standard curve for *R. dentocariosa*/*R. aeria*

The optimised cycling protocol for all six qPCR assays is summarised in Table 3. The final results are displayed in Fig. 2, with all primer tests conducted on the Quantstudio 6 Flex displayed in Supplement 3.

**Table 3:** Optimised cycling protocol for qPCR assays

Assay	Cycle type	Polymerase activation (1 cycle)		Amplification				
				Cycles	Denaturation		Annealing/Extension	
*S. mutans*	Standard	95 °C	3:00	40	95 °C	0:15	60 °C	1:00
*S. wiggsiae*	Standard	95 °C	3:00	40	95 °C	0:15	63 °C*	1:00
*P. gingivalis*	Fast	95 °C	3:00	40	95 °C	0:05	60 °C	0:30
*C. durum*	Fast	95 °C	3:00	40	95 °C	0:05	60 °C	0:30
*F. nucleatum* subsp. *fusiforme*	Standard	95 °C	3:00	40	95 °C	0:15	60.7 °C*	1:00
*R. dentocariosa*/*R. aeria*	Standard	95 °C	3:00	40	95 °C	0:15	60 °C	1:00

All times noted as min:sec; *optimal annealing temperature as determined by gradient PCR on CFX-384

Results for Primer test 1 are shown in Fig. 2 Panels 1–3 for optimised assays. The *P. gingivalis* (*R* = 0.996, Eff = 94.04%)*,* and *C. durum* assays (*R* = 0.998, Eff = 91.43%) worked, with excellent precision (*R*^2^ > 0.998) and efficiency within 90–110%, and the *F. nucleatum* subsp. *fusiforme* assay had efficiency of approximately 88% (*R* > 0.998). The *P. gingivalis* assay showed positive readings for two of the NTCs and so the assay was repeated with clean NTCs (Fig. 2 Panel 2; and Supplement 3), however with acceptable efficiency of 107% (*R* = 0.995) only when the lowest two dilutions (10^0, 10^1) and highest dilution (10^10) was omitted as the complete standard curve had an efficiency of 144%, with efficiency of 117% when only the highest and lowest dilutions (10^0, 10^10) were omitted. The *S. mutans* and *S. wiggsiae* assays showed no amplification, so the qPCR products from the mid-point dilutions of gBlocks standards (18,903.5 and 12,128.6 gene copies/µL respectively) were run on gel electrophoresis (Tapestation) and the results suggested that no products were formed from the qPCR reaction (Supplement 4). The primer-dimer reports for both failed assays were unremarkable and further testing for iatrogenic error was performed (Primer test 2).

Results for Primer test 2 showed that the *S. wiggsiae* assay signal on the RotorGene Q was too low. There was still amplification, but it did not cross 1 unit of fluorescence, which suggested the assay would not work well under current cycling conditions. The amplification of both assays was very low. To provide context, the Rotor Gene Q is capable of calibration to optimise low signals/fluorescence from the assays, and the *S. mutans* assay had low signal (up to 30 units) but still crossed the “threshold” line. The larger Quantstudio 6 Flex machine does not have the ability to optimise for low signal like the smaller RotorGene Q machine, so an additional primer test on the Quantstudio 6 Flex was still required to check this (Primer test 3). Therefore, preparations were made to run *S. wiggsiae* on Primer test 3, but with the primer set only (no probe) and with QuantiNova SYBR Green mastermix (Qiagen; Lot 169,046,282) with a melt curve.

Results for Primer test 3 (Supplement 3): the *S. mutans* assay with the probe, seemed to display good amplification and no cross reactivity with the *S. wiggsiae* gBlocks as the test for negative control (*R*^2^ = 0.999 Efficiency = 91.111%). For the *S. wiggsiae* assay run with SYBR intercalating dye and primers only, when all points were included, the efficiency was 87.1%. However, when the four outliers were omitted, the efficiency was 79% (exclude 1.2 × 10^10, and some of the replicates for the lowest three dilutions). Additionally, there was cross reactivity with the *S. mutans* gBlocks after Ct/Cq values of 30. This suggested the assay was not optimised for a specific primer set to use without the probe at 60 °C. For *S. wiggsiae* assays, all No Template Controls (NTC) had amplification due to the presence of primer-dimers when run at an annealing temperature of 60 °C. The calculated optimal annealing temperature (OAT) for the *S. wiggsiae* assay was 62 °C.

Results for Primer test 4 (Supplement 3 Figs. 1, 2) showed that the OAT was 63 °C for *S. wiggsiae* and 60.7 °C for *F. nucleatum subsp. fusiforme*. Results for Primer test 5 are shown in Fig. 2 Panels 4–5. Both the *S. wiggsiae* (*R* = 0.991, Eff = 90.32%) and *F. nucleatum* (*R* = 0.994, Eff = 87.14%) assays had good efficiency. When comparing the *F. nucleatum* subsp. *fusiforme* assay results from Primer test 1 (60 °C annealing temperature) with the current primer test (60.7 °C annealing temperature) (Supplement 3), it was noted that the assay had efficiencies approaching 90%, however, gene copy readings for sample A19 (an impure *S. sanguinis* culture in BHI) were in the order of 10^3 gene copies per reaction, while “indeterminate” readings were returned for sample B19 which was the same volume of sample as A19 but spiked onto an FTA card. As background, for other assays an approximately five to ten-fold decreased was generally noted between samples A19 and B19, with several other assays giving single gene copy numbers. Therefore, this observation for the *F. nucleatum* subsp. *fusiforme* assay was most unusual.

Results for Primer test 6 are shown in Fig. 2 Panel 6. The *S. mutans* assay generally worked well (*R* = 0.998, Eff = 94.07%), although single gene copy numbers were noted in the test samples (samples labelled A19 and DX swab) (Supplement 3).

Results for Primer test 7 are shown in Fig. 2 Panel 7 (or Supplement 3). The *R. dentocariosa* assay displayed good efficiency (*R* = 0.962, Eff = 93.1%).

### LOD/LOQ calculations

LOD and LOQ calculation results are displayed in Table 4.

**Table 4:** Limit of detection and limit of quantitation as calculated using R custom scripts published in Klymus et al. (2020) †

Assay	R.squared	Slope	Intercept	Low.95	LOD	LOQ	rep2.LOD	rep3.LOD	rep4.LOD	rep5.LOD	rep8.LOD
*S. mutans*				18.904	Unreliable						
*S. wiggsiae*	1	− 4.0110574	42.2442843	12.129	3.30753762	49	1.65382691	1.10255706	0.82691944	0.66153624	0.41346072
*P. gingivalis*				1.92	< 1.92 copies/reaction						
*F. nucleatum*				11.261	< 11.261 copies/reaction						
*C. durum*				12.833							
*R. dentocariosa*/*R. aeria*	1	− 3.0189925	41.195317	17.428	4.752704453	6093	2.37643504	1.58429828	1.18822606	0.95058183	0.59411445

R.squared: The R-squared value of linear regression of all standards Cq-values vs log10 of the starting quantities

Slope: The slope of the linear regression

Intercept: The y-intercept of the linear regression

Low.95: The lowest standard with at least 95% positive detection

LOD: The 95% limit of detection as determined by probit modelling

LOQ: The limit of quantification as determined by decay modelling, using the user-selected CV threshold of 0.35

^†^Klymus, K. E., Merkes, C. M., Allison, M. J., Goldberg, C. S., Helbing, C. C., Hunter, M. E., Richter, C. A. (2020). Reporting the limits of detection and quantification for environmental DNA assays. Environmental DNA, 2(3), 271–282. doi: 10.1002/edn3.29

While optimised assays generally showed good efficiency in the range of 90–100% with the exception of *F. nucleatum subsp. fusiforme*, many of the test samples swabbed from relatively healthy mouths, contained single gene copies for several assays.

### Verification of PCR products

Results for Sanger sequencing are shown in Supplement 5. The forward and reverse Sanger sequencing results, when combined, matched the sequence of the target sequences which the exception of a number of base pairs at either end of the amplicon.

## Discussion

This is the first study to detail qPCR optimisation procedures for dental plaque samples collected on FTA cards, which allow room temperature storage when cold storage is not feasible or when data collection involves extensive travel. The study confirmed that samples of dental plaque stored on FTA cards, were suitable for use in qPCR tests, and that a modified Powersoil DNA extraction protocol provided the best DNA yield and quality. The qPCR assays and synthetic standards that were designed and optimised, cover organisms associated with presence of dental disease, dental health, gingival health, and gingival disease. It was intended that the species-specific assays *S. mutans, S. wiggsiae, P. gingivalis, C. durum* and *R. dentocariosa/R. aeria* would allow detection using real time qPCR with high specificity.

While the use of proprietary qPCR assays made has provided much convenience, significant supplier-related delays related to the COVID-19 pandemic precluded their use, especially given a fixed travelling schedule for interstate data collection. Custom assays had a two-week turnaround time to delivery as opposed to two months for the proprietary assays. Conducting multiple sequence alignment and in silico design of species-specific assays is more time-consuming, however, the user has more control over performance of the assays and can verify their efficiency. Additionally, the MIQE guidelines for publication of qPCR data specify publication of sequences and efficiency data as essential and this is not possible with use of proprietary assays.

Four of the assays (*S. wiggsiae, C. durum, P. gingivalis, and F. nucleatum* subsp. *fusiforme*) were run with at least three biological replicates in triplicate, with two (*S. mutans, R. dentocariosa*) run with five biological replicates which included a COPAN FLOQswab and its corresponding FTA sample. All assays were run with samples A19, B19 and C19, which included an identical sample that was cultured BHI and on an FTA card. It was generally observed that there was up to ten-fold decrease in the gene copy numbers on the FTA cards when comparing this to the swabs. However, this was not the case for two of the six assays. Firstly, the sample labelled A19 showed gene copy numbers in the order of almost 10^3 for the *F. nucleatum* assay but with indeterminate readings on the corresponding FTA card sample, and this observation was replicated in both runs at 60* °C* and 60.7* °C* annealing temperature. It is hypothesised that the microstructural properties of *F. nucleatum* may preclude its extraction from the FTA card due to its abundance of sticky adhesins (Han et al. 2005; Muchova et al. 2022). Separately, it is noted that other researchers have observed issues with *F. nucleatum* for checkerboard analysis (Benn et al. 2022) but their observation may be linked to lack of specificity due to its similarity with other strains of *F. nucleatum* (Socransky et al. 2004). Overall, the *F. nucleatum* subsp. *fusiforme* assay in this body of work was optimised to an efficiency within 0.5% of the industry threshold of 90%, and while it may not be suited to use with FTA card samples, it may probably be acceptable to use with other standard nylon swabs if other assays cannot be found with no off-targets; this should be verified through further work. It is noteworthy to mention that the publication of the primer set (Shin et al. 2010) was published before *F. nucleatum subsp. fusiforme* was considered to be related to *F. nucleatum subsp. vincentii* some years later.

The *R. dentocariosa/R. aeria* assay was the second assay which showed a departure from the general observation of a ten-fold decrease between A19 and B19 samples or from corresponding swab to FTA card samples. For this assay, fewer gene copies were noted on the (culture in BHI) A19 sample than sample B19 on the FTA card. It may be that the microstructural properties of the bacteria make it more susceptible to DNA degradation during the vortex mixing step of the Powersoil protocol, whereas it may not have been as affected with the same degree of agitation of the FTA card during vortexing. Perhaps this theory could be tested by reducing the vortex mixing time if using culture in Powersoil reaction, although this may have limited applicability to this clinical situation where samples would be expected to be on swabs as opposed to culture.

With the exception of the *F. nucleatum* subsp. *fusiforme* and *R. dentocariosa/R. aeria* assays, most assays showed low or even single gene copy numbers on the test samples, especially *S. mutans* and *S. wiggsiae.* This may have occurred as the test samples were mostly swabbed from relatively healthy subjects. Additionally, for the re-run of *P. gingivali*s which was performed some months after the initial test run, observed gene copy counts for biological replicates were up to one-tenth of the observed amounts in Primer test 1, suggesting DNA degradation. The discrepancies observed in the efficiency of the standard curve between the initial and the repeat runs of *P. gingivalis* may have arisen due to pipetting error, despite use of a pipetting robot. Alternatively, the TE buffer was used in the resuspension protocol and the EDTA may have acted as an inhibitor; this may be unlikely as the efficiency was not affected during the first test run. While it may have been ideal, it was not feasible to use DNA template from samples collected from the Rett syndrome subjects in the overall multicentre study due to only 50 µL being available for limited runs of assays. In several assays, gene copy numbers were less than the limit of quantification (LOQ) or outside the range of the standard curve, and so there is merit in optimising the same qPCR assays for use in digital droplet PCR instruments where standard curves are not required due to calculations being performed at the end point as opposed to in “real time” as with qPCR, thus minimising potential effect of inhibitors. This would allow more validity for an experiment if one was expecting low gene copy numbers as with the intended clinical study due to a) the nature of swabbing procedure on those with severe intellectual disability and b) expected loss of sample on FTA card, which was not as obvious when assessing DNA concentration following DNA extraction, but more noticeable when during qPCR optimisation.

With the limitations of expected low DNA yield and a 50 µL elution volume of extracted DNA, the lowest reaction volume was chosen (2ul) in line with the parameters outlined in the manufacturer mastermix protocol. The disadvantage of using little DNA template was the increased likelihood of subsampling error particularly in the presence of low gene copies. If future optimisation of these qPCR assays is conducted on a dPCR instrument, then issues with potential subsampling error should be explored during dPCR optimisation by testing singleplex assays at 2 µL, 4 µL, and 6 µL and also through a trial of duplexing of different combinations of assays with SUN fluorophore an assay with a FAM fluorophore. If multiplexing is successful on a dPCR machine, then it would be best to increase the DNA template amount given the finite volume of precious DNA samples.

## Conclusion

The present work details a novel approach to sample collection and DNA extraction from dental plaque when cold storage is not feasible, using FTA cards. Additionally, this work outlines the design and validation of assays and synthetic standards for absolute quantitation using real time qPCR. With the exception of *F. nucleatum subsp. fusiforme,* most assays, namely *S. mutans, S. wiggsiae, C. durum, R. dentocariosa,* and *P. gingivalis,* allowed detection using real time qPCR with high specificity on samples collected on FTA cards. Assays generally had good efficiency. However, for several assays, low gene copy numbers in test samples were lower than the LOQ. Future work could explore optimisation of the same qPCR assays for use with dPCR.

### Supplementary Information

Below is the link to the electronic supplementary material.

Supplementary file1 (PDF 1647 KB)

## References

[CR2] AlEraky DM, Madi M, El Tantawi M, AlHumaid J, Fita S, AbdulAzeez S, Borgio JF, Al-Harbi FA, Alagl AS. Predominance of non-Streptococcus mutans bacteria in dental biofilm and its relation to caries progression. Saudi J Biol Sci. 2021;28(12):7390–5. 10.1016/j.sjbs.2021.08.052. 34867042 10.1016/j.sjbs.2021.08.052 PMC8626303

[CR3] American Academy of Pediatric Dentistry. (2022). Caries-risk assessment and management for infants, children, and adolescents. *The Reference Manual of Pediatric Dentistry.*Chicago, IL. American Academy of Pediatric Dentistry; 2023:301–307

[CR4] American Dental Association. (2023a). Caries Risk Assessment Form (Age 0–6). Retrieved from https://www.ada.org/-/media/project/ada-organization/ada/ada-org/files/resources/library/oral-health-topics/ada_caries_risk_assessment.pdf

[CR5] American Dental Association. (2023b). Caries Risk Assessment Form (Age >6). Retrieved from https://www.ada.org/-/media/project/ada-organization/ada/ada-org/files/resources/library/oral-health-topics/topic_caries_over6.pdf

[CR6] Amir R, Van den Veyver I, Wan M, Tran C, Francke U, Zoghbi H. Rett syndrome is caused by mutations in X-linked MECP2, encoding methyl-CpG-binding protein 2. Nat Genet. 1999;23(2):185–8. 10.1038/13810. 10508514 10.1038/13810

[CR7] Ammann TW, Bostanci N, Belibasakis GN, Thurnheer T. Validation of a quantitative real-time PCR assay and comparison with fluorescence microscopy and selective agar plate counting for species-specific quantification of an in vitro subgingival biofilm model. J Periodontal Res. 2013;48(4):517–26. 10.1111/jre.12034. 23278531 10.1111/jre.12034

[CR8] Avila-Campos MJ, Sacchi CT, Whitney AM, Steigerwalt AG, Mayer LW. Arbitrarily primed-polymerase chain reaction for identification and epidemiologic subtyping of oral isolates of *Fusobacterium nucleatum*. J Periodontol. 1999;70(10):1202–8. 10.1902/jop.1999.70.10.1202. 10534075 10.1902/jop.1999.70.10.1202

[CR9] Benn AML, Heng NCK, Thomson WM, Sissons CH, Gellen LS, Gray AR, Broadbent JM. Associations of sex, oral hygiene and smoking with oral species in distinct habitats at age 32 years. Eur J Oral Sci. 2022;130(1): e12829. 10.1111/eos.12829. 34874583 10.1111/eos.12829

[CR10] Bustin SA, Benes V, Garson JA, Hellemans J, Huggett J, Kubista M, Mueller R, Nolan T, Pfaffl MW, Shipley GL, Vandesompele J, Wittwer CT. The MIQE guidelines: minimum information for publication of quantitative real-time PCR experiments. Clin Chem. 2009;55(4):611–22. 10.1373/clinchem.2008.112797. 19246619 10.1373/clinchem.2008.112797

[CR11] Da Silva SN, Gimenez T, Souza RC, Mello-Moura ACV, Raggio DP, Morimoto S, Lara JS, Soares GC, Tedesco TK. Oral health status of children and young adults with autism spectrum disorders: systematic review and meta-analysis. Int J Pediatr Dent. 2017;27(5):388–98. 10.1111/ipd.12274. 10.1111/ipd.12274 27796062

[CR12] Dieguez-Perez M, De Nova-Garcia MJ, Mourelle-Martinez MR, Bartolome-Villar B. Oral health in children with physical (cerebral palsy) and intellectual (Down syndrome) disabilities: systematic review I. J Clin Exp Dent. 2016;8(3):e337–43. 10.4317/jced.52922. 27398187 10.4317/jced.52922 PMC4930646

[CR13] Dinakaran V, Mandape SN, Shuba K, Pratap S, Sakhare SS, Tabatabai MA, Smoot DT, Farmer-Dixon CM, Kesavalu LN, Adunyah SE, Southerland JH, Gangula PR. Identification of specific oral and gut pathogens in full thickness colon of colitis patients: implications for colon motility. Front Microbiol. 2018;9:3220. 10.3389/fmicb.2018.03220. 30666239 10.3389/fmicb.2018.03220 PMC6330997

[CR14] Fehr S, Bebbington A, Nassar N, Downs J, Ronen GM, de Klerk N, Leonard H. Trends in the diagnosis of Rett syndrome in Australia. Pediatr Res. 2011;70(3):313–9. 10.1203/PDR.0b013e3182242461. 21587099 10.1203/PDR.0b013e3182242461 PMC3152673

[CR15] Fuertes-Gonzalez MC, Silvestre FJ. Oral health in a group of patients with Rett syndrome in the regions of Valencia and Murcia (Spain): a case-control study. Med Oral Patol Oral Y Cir Bucal. 2014;19(6):E598–604. 10.4317/medoral.19743. 10.4317/medoral.19743 PMC4259377 25350594

[CR16] Gharbia SE, Shah HN, Lawson PA, Haapasalo M. The distribution and frequency of *Fusobacterium nucleatum* subspecies in the human oral cavity. Oral Microbiol Immunol. 1990;5(6):324–7. 10.1111/j.1399-302X.1990.tb00434.x. 2098710 10.1111/j.1399-302X.1990.tb00434.x

[CR17] Han YW, Ikegami A, Rajanna C, Kawsar HI, Zhou Y, Li M, Sojar HT, Genco RJ, Kuramitsu HK, Deng CX. Identification and characterization of a novel adhesin unique to oral fusobacteria. J Bacteriol. 2005;187(15):5330–40. 10.1128/jb.187.15.5330-5340.2005. 16030227 10.1128/jb.187.15.5330-5340.2005 PMC1196005

[CR18] Isaac RD, Sanjeev K, Subbulakshmi CL, Amirtharaj LV, Sekar M. Identification of a novel bacterium *Scardovia wiggsiae* in high caries risk adolescence: a metagenomic and melt curve analysis. J Conserv Dent. 2022;25(3):297–305. 10.4103/jcd.jcd_79_22. 35836558 10.4103/jcd.jcd_79_22 PMC9274687

[CR19] Kim M-K, Kim H-S, Kim B-O, Yoo S-Y, Seong J-H, Kim D-K, Lee S-E, Choe S-J, Park J-C, Min B-M. Multiplex PCR using conserved and species-specific 16S rDNA primers for simultaneous detection of *Fusobacterium nucleatum* and Actinobacillus actinomycetemcomitans. J Microbiol Biotechnol. 2004;14(1):110–5. 10.4014/jmb.2108.08016

[CR20] Klymus KE, Merkes CM, Allison MJ, Goldberg CS, Helbing CC, Hunter ME, Jackson CA, Lance RF, Mangan AM, Monroe EM, Piaggio AJ, Stokdyk JP, Wilson CC, Richter CA. Reporting the limits of detection and quantification for environmental DNA assays. Environ DNA. 2020;2(3):271–82. 10.1002/edn3.29. 10.1002/edn3.29

[CR22] Lai YYL, King NM, Downs J, Leonard H. Management of oral and dental problems in Rett syndrome: a narrative review of the literature. J Disabil Oral Health. 2018a;19(2):39–48.

[CR21] Lai Y, Wong K, King NM, Downs J, Leonard H. Oral health experiences of individuals with Rett syndrome: a retrospective study. BMC Oral Health. 2018b. 10.1186/s12903-018-0651-y. 30497449 10.1186/s12903-018-0651-y PMC6267076

[CR23] Lai YYL, Downs J, Zafar S, Wong K, Walsh L, Leonard H. Oral health care and service utilisation in individuals with Rett syndrome: an international cross-sectional study. J Intellect Disabil Res. 2021;65(6):561–76. 10.1111/jir.12834. 33764620 10.1111/jir.12834

[CR24] Lai Y, Downs JA, Wong K, Zafar S, Walsh LJ, Leonard HM. Enablers and barriers in dental attendance in Rett syndrome: an international observational study. Spec Care Dentist. 2022;42(6):565–74. 10.1111/scd.12712. 35290682 10.1111/scd.12712 PMC9790614

[CR25] Lai YYL, Downs JA, Wong K, Zafar S, Walsh LJ, Leonard HM. Oral parafunction and bruxism in Rett syndrome and associated factors: an observational study. Oral Dis. 2023;29(1):220–31. 10.1111/odi.13924. 34033206 10.1111/odi.13924

[CR26] Lam PP, Du R, Peng S, McGrath CP, Yiu CK. Oral health status of children and adolescents with autism spectrum disorder: a systematic review of case-control studies and meta-analysis. Autism. 2020;24(5):1047–66. 10.1177/1362361319877337. 31931609 10.1177/1362361319877337

[CR27] Lochman J, Zapletalova M, Poskerova H, Izakovicova Holla L, Borilova Linhartova P. Rapid multiplex real-time PCR method for the detection and quantification of selected cariogenic and periodontal bacteria. Diagnostics. 2019;10(1):8. 10.3390/diagnostics10010008. 31877891 10.3390/diagnostics10010008 PMC7168300

[CR28] Lourenço TGB, Heller D, Silva-Boghossian CM, Cotton SL, Paster BJ, Colombo APV. Microbial signature profiles of periodontally healthy and diseased patients. J Clin Periodontol. 2014;41(11):1027–36. 10.1111/jcpe.12302. 25139407 10.1111/jcpe.12302 PMC4213353

[CR29] Miles M, Saul D (2015) Improved elution of DNA from Whatman FTA™ cards using prepGEM/forensicGEM Universal extraction kits. Retrieved from https://microgembio.com/m_resource/improved-elution-of-dna-from-whatman-fta-cards-using-prepgem-forensicgem-universal-extraction-kits

[CR30] Muchova M, Balacco DL, Grant MM, Chapple ILC, Kuehne SA, Hirschfeld J. Fusobacterium nucleatum subspecies differ in biofilm forming ability in vitro. Front Oral Health. 2022;3: 853618. 10.3389/froh.2022.853618. 35368312 10.3389/froh.2022.853618 PMC8967363

[CR31] Neul JL, Kaufmann WE, Glaze DG, Christodoulou J, Clarke AJ, Bahi-Buisson N, Leonard H, Bailey ME, Schanen NC, Zappella M, Renieri A, Huppke P, Percy AK, RettSearch Consortium. Rett syndrome: revised diagnostic criteria and nomenclature. Ann Neurol. 2010;68(6):944–50. 10.1002/ana.22124. 21154482 10.1002/ana.22124 PMC3058521

[CR32] New England Biolabs. Protocol for Genomic DNA Cleanup (NEB #T3010). Retrieved from https://www.nebiolabs.com.au/protocols/2018/10/25/protocol-for-genomic-dna-cleanup-t3010

[CR33] Park S-N, Lim YK, Kook J-K. Development of quantitative real-time PCR primers for detecting 42 oral bacterial species. Arch Microbiol. 2013;195(7):473–82. 10.1007/s00203-013-0896-4. 23689247 10.1007/s00203-013-0896-4

[CR34] Philip N, Suneja B, Walsh L. Beyond Streptococcus mutans: clinical implications of the evolving dental caries aetiological paradigms and its associated microbiome. Br Dent J. 2018;224(4):219–25. 10.1038/sj.bdj.2018.81. 29449651 10.1038/sj.bdj.2018.81

[CR35] Ruiz L-A, Diniz M-B, Loyola-Rodriguez J-P, Habibe C-H, Garrubbo C-C, Santos M-T-B-R. A controlled study comparing salivary osmolality, caries experience and caries risk in patients with cerebral palsy. Med Oral Patol Oral y Cir Bucal. 2018;23(2):e211-215. 10.4317/medoral.22135. 10.4317/medoral.22135 PMC5911353 29476677

[CR36] Sakamoto M, Takeuchi Y, Umeda M, Ishikawa I, Benno Y. Rapid detection and quantification of five periodontopathic bacteria by real-time PCR. Microbiol Immunol. 2001;45(1):39–44. 10.1111/j.1348-0421.2001.tb01272.x. 11270605 10.1111/j.1348-0421.2001.tb01272.x

[CR37] Santos MTBR, Ferreira MCD, Mendes FM, de Oliveira Guaré R. Assessing salivary osmolality as a caries risk indicator in cerebral palsy children. Int J Pediatr Dent. 2014;24(2):84–9. 10.1111/ipd.12030. 10.1111/ipd.12030 23551764

[CR38] Shin HS, Kim M-J, Kim H-S, Park S-N, Kim DK, Baek D-H, Kim C, Kook J-K. Development of strain-specific PCR primers for the identification of *Fusobacterium nucleatum* subsp fusiforme ATCC 51190T and subsp vincentii ATCC 49256T. Anaerobe. 2010;16(1):43–6. 10.1016/j.anaerobe.2009.04.003. 19398030 10.1016/j.anaerobe.2009.04.003

[CR1] Sigma Aldrich . (2020). Whatman® FTA® card technology. Retrieved from https://www.sigmaaldrich.com/AU/en/product/sigma/whawb120205

[CR39] Socransky SS, Haffajee AD, Smith C, Martin L, Haffajee JA, Uzel NG, Goodson JM. Use of checkerboard DNA-DNA hybridization to study complex microbial ecosystems. Oral Microbiol Immunol. 2004;19(6):352–62. 10.1111/j.1399-302x.2004.00168.x. 15491460 10.1111/j.1399-302x.2004.00168.x

[CR40] Tanner ACR, Kent RL Jr, Holgerson PL, Hughes CV, Loo CY, Kanasi E, Chalmers NI, Johansson I. Microbiota of severe early childhood caries before and after therapy. J Dent Res. 2011;90(11):1298–305. 10.1177/0022034511421201. 21868693 10.1177/0022034511421201 PMC3188461

[CR41] Topcuoglu N, Paltura C, Kulekci M, Ustek D, Kulekci G. Real-time polymerase chain reaction versus conventional PCR: a comparison between two methods for the detection of *Fusobacterium nucleatum* in saliva, nasopharyngeal secretion and middle ear effusion samples. Biotechnol Biotechnol Equip. 2013;27(3):3825–8. 10.5504/bbeq.2013.0022. 10.5504/bbeq.2013.0022

[CR42] Tran S, Rudney J. Improved multiplex PCR using conserved and species-specific 16S rRNA gene primers for simultaneous detection of *Actinobacillus actinomycetemcomitans*, *Bacteroides forsythus*, and *Porphyromonas gingivalis*. J Clin Microbiol. 1999;37(11):3504–8. 10.1128/JCM.37.11.3504-3508.1999. 10523542 10.1128/JCM.37.11.3504-3508.1999 PMC85679

[CR43] Yeung DF, Parsa A, Wong JC, Chatur N, Salh B. A case of *Rothia dentocariosa* bacteremia in a patient receiving infliximab for ulcerative colitis. Am J Gastroenterol. 2014;109(2):297–8. 10.1038/ajg.2013.366. 24496430 10.1038/ajg.2013.366

[CR44] Yoshida A, Suzuki N, Nakano Y, Kawada M, Oho T, Koga T. Development of a 5′ nuclease-based real-time PCR assay for quantitative detection of cariogenic dental pathogens *Streptococcus mutans* and *Streptococcus sobrinus*. J Clin Microbiol. 2003;41(9):4438–41. 10.1128/jcm.41.9.4438-4441.2003. 12958287 10.1128/jcm.41.9.4438-4441.2003 PMC193796

