# Supplementary material for: qPCR assay optimisation for a clinical study comparing oral health risk in Rett syndrome
Source: Eur Arch Paediatr Dent. 2024 Jun 26;25(4):547–60. doi: 10.1007/s40368-024-00912-8 (PMC11341660; doi:10.1007/s40368-024-00912-8)
Supplement: Supplementary file 1 — Supplementary file1 (PDF 1647 KB) [file 40368_2024_912_MOESM1_ESM.pdf]

Supplement 1.

Supplement 1 Table 1. Methodological approach to recruitment targets of case/sibling control dyads

**State**	**SA**	**WA**	**Qld**
Total RTT families in state†	22	35	64
Reference city	Adelaide	Perth	Brisbane
CBD/within 1-1.5 hrs drive from city centre	18	31	45 ‡
Conservative Target recruitment number of case/sibling control dyads (~50% of families in CBD area)	11 §	16	22

†of genetically confirmed cases of RTT who are alive

‡includes 2 families living near QLD/NSW border

§actual recruited number- SA pilot data collection completed

Supplement 2

Supplement 2 Table-1 Reaction setup for Primer test 2 †

	S. mutans Probe	S. wiggsiae Probe
S. mutans standards	18903502.17 gene copies	18903502.17 gene copies
	1890350.217 gene copies	1890350.217 gene copies
	189035.0217 gene copies	189035.0217 gene copies
	No Template Control	No Template Control
S. wiggsiae standards	12128592.98 gene copies	12128592.98 gene copies
	1212859.298 gene copies	1212859.298 gene copies
	121285.9298 gene copies	121285.9298 gene copies

†Reactions were run in duplicate in 28 wells of 10ul on a RotorGene Q 5-Plex HRM (Qiagen)

Supplement 2 Table 2 DNA extraction results from DNA extraction

Extraction Method	Sample ID	Area of FTA card used	Nanodrop ng/uL	A260/A280	A260/A230	A260	A280	Qubit reading ng/uL
1	YL FTA	30mm diameter	22.2	0.49	0.12	0.44	0.91	0.178
1	ID240	2 x 2mm diameter	2.3	1.1	0.18	0.05	0.04	0.0704
1	ID261	2 x 2mm diameter	2.3	0.67	0.11	0.05	0.07	0.0202
2	YL FTA	30mm diameter	8.3	0.6	0.1			0.129
3‡	YL FTA	2 mm diameter	n/a†	n/a†	n/a†	n/a†	n/a†	0.1
4	A19= S sanguinis broth	Entire sample	9.4	1.76	1.99	0.19	0.11	4.12
4	B19= S sanguinis FTA	Entire sample	4.2	1.64	1.69	0.08	0.05	2.03
4	C19=YL FTA	Entire sample	1.9	1.47	2.26	0.04	0.03	Out of range
4‡	DX swab	Entire sample	n/a†	n/a†	n/a†	n/a†	n/a†	1.45
4‡	DX FTA	Entire sample	n/a†	n/a†	n/a†	n/a†	n/a†	1.35

Legend:
Extraction Method 1= modified Whatman Method B protocol using alkali lysis
Extraction Method 2= modified Whatman Method B (alkali lysis) with repurification and ethanol precipitation
Extraction Method 3= Microgem Bio Universal Kit
Extraction Method 4= modified Powersoil protocol
†not provided by external laboratory
‡Performed at external lab Australian Genome Research Facility Brisbane laboratory

Supplement 3

Supplement 3Table 1 Complete list of qPCR optimisation test results run on Quantstudio 6 Flex (see next page)

Primer test	Sample Name	Target Name	CT	Quantity
1	NTC	C durum spp 16s	Undetermined	
1	NTC	C durum spp 16s	Undetermined	
1	NTC	C durum spp 16s	Undetermined	
1		C durum spp 16s	4.124	12833183744
1		C durum spp 16s	4.136	12833183744
1		C durum spp 16s	4.045	12833183744
1		C durum spp 16s	6.861	1283318400
1		C durum spp 16s	6.755	1283318400
1		C durum spp 16s	6.817	1283318400
1		C durum spp 16s	11.169	128331832
1		C durum spp 16s	10.805	128331832
1		C durum spp 16s	10.865	128331832
1		C durum spp 16s	15.212	12833184
1		C durum spp 16s	14.911	12833184
1		C durum spp 16s	15.089	12833184
1		C durum spp 16s	18.287	1283318.375
1		C durum spp 16s	18.527	1283318.375
1		C durum spp 16s	18.441	1283318.375
1		C durum spp 16s	21.398	128331.836
1		C durum spp 16s	21.513	128331.836
1		C durum spp 16s	21.566	128331.836
1		C durum spp 16s	25.959	12833.184
1		C durum spp 16s	25.188	12833.184
1		C durum spp 16s	25.119	12833.184
1		C durum spp 16s	28.486	1283.318
1		C durum spp 16s	28.655	1283.318
1		C durum spp 16s	28.65	1283.318
1		C durum spp 16s	31.715	128.332
1		C durum spp 16s	31.654	128.332
1		C durum spp 16s	31.973	128.332
1		C durum spp 16s	36.745	12.833
1		C durum spp 16s	36.489	12.833
1		C durum spp 16s	36.429	12.833
1		C durum spp 16s	Undetermined	1.283
1		C durum spp 16s	Undetermined	1.283
1		C durum spp 16s	38.258	1.283
1	Sample 1 - A19	C durum spp 16s	33.799	48.265
1	Sample 1 - A19	C durum spp 16s	32.995	81.351
1	Sample 1 - A19	C durum spp 16s	33.966	43.315
1	Sample 2 - B19	C durum spp 16s	37.175	5.391
1	Sample 2 - B19	C durum spp 16s	36.793	6.907
1	Sample 2 - B19	C durum spp 16s	38.109	2.939
1	Sample 3 - C19	C durum spp 16s	33.976	43.031
1	Sample 3 - C19	C durum spp 16s	33.306	66.463
1	Sample 3 - C19	C durum spp 16s	33.315	66.106
1	NTC	F nucleatum subspp fusiforme	Undetermined	
1	NTC	F nucleatum subspp fusiforme	Undetermined	
1	NTC	F nucleatum subspp fusiforme	Undetermined	
1		F nucleatum subspp fusiforme	10.904	112614048
1		F nucleatum subspp fusiforme	10.665	112614048
1		F nucleatum subspp fusiforme	10.724	112614048
1		F nucleatum subspp fusiforme	14.522	11261405
1		F nucleatum subspp fusiforme	14.542	11261405
1		F nucleatum subspp fusiforme	14.697	11261405
1		F nucleatum subspp fusiforme	17.915	1126140.5
1		F nucleatum subspp fusiforme	18.173	1126140.5
1		F nucleatum subspp fusiforme	17.947	1126140.5
1		F nucleatum subspp fusiforme	22.227	112614.047
1		F nucleatum subspp fusiforme	21.972	112614.047
1		F nucleatum subspp fusiforme	22.153	112614.047
1		F nucleatum subspp fusiforme	25.288	11261.404
1		F nucleatum subspp fusiforme	25.448	11261.404
1		F nucleatum subspp fusiforme	25.563	11261.404
1		F nucleatum subspp fusiforme	29.09	1126.141
1		F nucleatum subspp fusiforme	29.338	1126.141
1		F nucleatum subspp fusiforme	29.226	1126.141
1		F nucleatum subspp fusiforme	32.744	112.614
1		F nucleatum subspp fusiforme	32.667	112.614
1		F nucleatum subspp fusiforme	32.879	112.614
1		F nucleatum subspp fusiforme	36.626	11.261
1		F nucleatum subspp fusiforme	35.515	11.261
1		F nucleatum subspp fusiforme	36.591	11.261
1	Sample 1 - A19	F nucleatum subspp fusiforme	29.441	913.528
1	Sample 1 - A19	F nucleatum subspp fusiforme	29.327	981.476
1	Sample 1 - A19	F nucleatum subspp fusiforme	29.553	851.356

1	Sample 2 - B19	F nucleatum subspp fusiforme	Undetermined	
1	Sample 2 - B19	F nucleatum subspp fusiforme	Undetermined	
1	Sample 2 - B19	F nucleatum subspp fusiforme	Undetermined	
1	Sample 3 - C19	F nucleatum subspp fusiforme	Undetermined	
1	Sample 3 - C19	F nucleatum subspp fusiforme	Undetermined	
1	Sample 3 - C19	F nucleatum subspp fusiforme	Undetermined	
1	NTC	P gingivalis spp hmuY	34.076	
1	NTC	P gingivalis spp hmuY	35.892	
1	NTC	P gingivalis spp hmuY	Undetermined	
1		P gingivalis spp hmuY	3.144	19198586880
1		P gingivalis spp hmuY	3.016	19198586880
1		P gingivalis spp hmuY	3.016	19198586880
1		P gingivalis spp hmuY	4.649	1919858688
1		P gingivalis spp hmuY	4.441	1919858688
1		P gingivalis spp hmuY	4.563	1919858688
1		P gingivalis spp hmuY	7.953	191985872
1		P gingivalis spp hmuY	8.136	191985872
1		P gingivalis spp hmuY	11.241	19198588
1		P gingivalis spp hmuY	11.345	19198588
1		P gingivalis spp hmuY	11.531	19198588
1		P gingivalis spp hmuY	15.343	1919858.75
1		P gingivalis spp hmuY	15.069	1919858.75
1		P gingivalis spp hmuY	15.163	1919858.75
1		P gingivalis spp hmuY	18.892	191985.875
1		P gingivalis spp hmuY	18.152	191985.875
1		P gingivalis spp hmuY	18.36	191985.875
1		P gingivalis spp hmuY	22.285	19198.588
1		P gingivalis spp hmuY	21.754	19198.588
1		P gingivalis spp hmuY	21.958	19198.588
1		P gingivalis spp hmuY	25.955	1919.859
1		P gingivalis spp hmuY	25.492	1919.859
1		P gingivalis spp hmuY	25.461	1919.859
1		P gingivalis spp hmuY	29.255	191.986
1		P gingivalis spp hmuY	29.402	191.986
1		P gingivalis spp hmuY	29.467	191.986
1		P gingivalis spp hmuY	34.23	19.199
1		P gingivalis spp hmuY	33.344	19.199
1		P gingivalis spp hmuY	32.509	19.199
1		P gingivalis spp hmuY	35.258	1.92
1		P gingivalis spp hmuY	37.489	1.92
1		P gingivalis spp hmuY	37.206	1.92
1	Sample 1 - A19	P gingivalis spp hmuY	35.624	2.933
1	Sample 1 - A19	P gingivalis spp hmuY	33.785	9.928
1	Sample 1 - A19	P gingivalis spp hmuY	34.306	7.03
1	Sample 2 - B19	P gingivalis spp hmuY	Undetermined	
1	Sample 2 - B19	P gingivalis spp hmuY	Undetermined	
1	Sample 2 - B19	P gingivalis spp hmuY	37.119	1.089
1	Sample 3 - C19	P gingivalis spp hmuY	Undetermined	
1	Sample 3 - C19	P gingivalis spp hmuY	Undetermined	
1	Sample 3 - C19	P gingivalis spp hmuY	35.005	4.422
1	NTC	S mutans UA159 gtfB	Undetermined	
1	NTC	S mutans UA159 gtfB	Undetermined	
1	NTC	S mutans UA159 gtfB	Undetermined	
1		S mutans UA159 gtfB	Undetermined	18903502848
1		S mutans UA159 gtfB	Undetermined	18903502848
1		S mutans UA159 gtfB	Undetermined	18903502848
1		S mutans UA159 gtfB	Undetermined	1890350208
1		S mutans UA159 gtfB	Undetermined	1890350208
1		S mutans UA159 gtfB	Undetermined	1890350208
1		S mutans UA159 gtfB	Undetermined	189035024
1		S mutans UA159 gtfB	Undetermined	189035024
1		S mutans UA159 gtfB	Undetermined	189035024
1		S mutans UA159 gtfB	Undetermined	18903502
1		S mutans UA159 gtfB	Undetermined	18903502
1		S mutans UA159 gtfB	Undetermined	18903502
1		S mutans UA159 gtfB	Undetermined	1890350.25
1		S mutans UA159 gtfB	Undetermined	1890350.25
1		S mutans UA159 gtfB	Undetermined	1890350.25
1		S mutans UA159 gtfB	Undetermined	189035.016
1		S mutans UA159 gtfB	Undetermined	189035.016
1		S mutans UA159 gtfB	Undetermined	189035.016
1		S mutans UA159 gtfB	Undetermined	18903.502
1		S mutans UA159 gtfB	Undetermined	18903.502
1		S mutans UA159 gtfB	Undetermined	18903.502
1		S mutans UA159 gtfB	Undetermined	1890.35
1		S mutans UA159 gtfB	Undetermined	1890.35

1		S mutans UA159 gtfB	Undetermined	1890.35
1		S mutans UA159 gtfB	Undetermined	189.035
1		S mutans UA159 gtfB	Undetermined	189.035
1		S mutans UA159 gtfB	Undetermined	189.035
1		S mutans UA159 gtfB	Undetermined	18.904
1		S mutans UA159 gtfB	Undetermined	18.904
1		S mutans UA159 gtfB	Undetermined	18.904
1		S mutans UA159 gtfB	Undetermined	1.89
1		S mutans UA159 gtfB	Undetermined	1.89
1		S mutans UA159 gtfB	Undetermined	1.89
1	Sample 1 - A19	S mutans UA159 gtfB	Undetermined	
1	Sample 1 - A19	S mutans UA159 gtfB	Undetermined	
1	Sample 1 - A19	S mutans UA159 gtfB	Undetermined	
1	Sample 2 - B19	S mutans UA159 gtfB	Undetermined	
1	Sample 2 - B19	S mutans UA159 gtfB	Undetermined	
1	Sample 2 - B19	S mutans UA159 gtfB	Undetermined	
1	Sample 3 - C19	S mutans UA159 gtfB	Undetermined	
1	Sample 3 - C19	S mutans UA159 gtfB	Undetermined	
1	Sample 3 - C19	S mutans UA159 gtfB	Undetermined	
1	NTC	S wiggsiae spp 16s	Undetermined	
1	NTC	S wiggsiae spp 16s	Undetermined	
1	NTC	S wiggsiae spp 16s	Undetermined	
1		S wiggsiae spp 16s	Undetermined	12128592896
1		S wiggsiae spp 16s	Undetermined	12128592896
1		S wiggsiae spp 16s	Undetermined	12128592896
1		S wiggsiae spp 16s	Undetermined	1212859264
1		S wiggsiae spp 16s	Undetermined	1212859264
1		S wiggsiae spp 16s	Undetermined	1212859264
1		S wiggsiae spp 16s	Undetermined	121285928
1		S wiggsiae spp 16s	Undetermined	121285928
1		S wiggsiae spp 16s	Undetermined	121285928
1		S wiggsiae spp 16s	Undetermined	12128593
1		S wiggsiae spp 16s	Undetermined	12128593
1		S wiggsiae spp 16s	Undetermined	12128593
1		S wiggsiae spp 16s	Undetermined	1212859.25
1		S wiggsiae spp 16s	Undetermined	1212859.25
1		S wiggsiae spp 16s	Undetermined	1212859.25
1		S wiggsiae spp 16s	Undetermined	121285.93
1		S wiggsiae spp 16s	Undetermined	121285.93
1		S wiggsiae spp 16s	Undetermined	121285.93
1		S wiggsiae spp 16s	Undetermined	12128.593
1		S wiggsiae spp 16s	Undetermined	12128.593
1		S wiggsiae spp 16s	Undetermined	12128.593
1		S wiggsiae spp 16s	Undetermined	1212.859
1		S wiggsiae spp 16s	Undetermined	1212.859
1		S wiggsiae spp 16s	Undetermined	1212.859
1		S wiggsiae spp 16s	Undetermined	121.286
1		S wiggsiae spp 16s	Undetermined	121.286
1		S wiggsiae spp 16s	Undetermined	121.286
1		S wiggsiae spp 16s	Undetermined	12.129
1		S wiggsiae spp 16s	Undetermined	12.129
1		S wiggsiae spp 16s	Undetermined	12.129
1		S wiggsiae spp 16s	Undetermined	1.213
1		S wiggsiae spp 16s	Undetermined	1.213
1		S wiggsiae spp 16s	Undetermined	1.213
1	Sample 1 - A19	S wiggsiae spp 16s	Undetermined	
1	Sample 1 - A19	S wiggsiae spp 16s	Undetermined	
1	Sample 1 - A19	S wiggsiae spp 16s	Undetermined	
1	Sample 2 - B19	S wiggsiae spp 16s	Undetermined	
1	Sample 2 - B19	S wiggsiae spp 16s	Undetermined	
1	Sample 2 - B19	S wiggsiae spp 16s	Undetermined	
1	Sample 3 - C19	S wiggsiae spp 16s	Undetermined	
1	Sample 3 - C19	S wiggsiae spp 16s	Undetermined	
1	Sample 3 - C19	S wiggsiae spp 16s	Undetermined	
3	NTC	S mutans UA159	Undetermined	
3	NTC	S mutans UA159	Undetermined	
3	NTC	S mutans UA159	Undetermined	
3		S mutans UA159	6.27	1890350208
3		S mutans UA159	6.211	1890350208
3		S mutans UA159	6.267	1890350208
3		S mutans UA159	9.761	189035024
3		S mutans UA159	9.718	189035024
3		S mutans UA159	9.831	189035024
3		S mutans UA159	13.275	18903502
3		S mutans UA159	13.249	18903502
3		S mutans UA159	13.252	18903502

3		S mutans UA159	17.112	1890350.25
3		S mutans UA159	17.077	1890350.25
3		S mutans UA159	17.106	1890350.25
3		S mutans UA159	20.399	189035.016
3		S mutans UA159	20.271	189035.016
3		S mutans UA159	20.404	189035.016
3		S mutans UA159	24.264	18903.502
3		S mutans UA159	24.195	18903.502
3		S mutans UA159	24.32	18903.502
3		S mutans UA159	27.361	1890.35
3		S mutans UA159	27.317	1890.35
3		S mutans UA159	27.242	1890.35
3		S mutans UA159	31.444	189.035
3		S mutans UA159	31.367	189.035
3		S mutans UA159	31.938	189.035
3		S mutans UA159	34	18.904
3		S mutans UA159	34.442	18.904
3		S mutans UA159	35.04	18.904
3		S mutans UA159	38.032	1.89
3		S mutans UA159	Undetermined	1.89
3		S mutans UA159	Undetermined	1.89
3	NTC	S wiggsiae 16S - primers	32.493	
3	NTC	S wiggsiae 16S - primers	33.448	
3	NTC	S wiggsiae 16S - primers	33.488	
3		S wiggsiae 16S - primers	Undetermined	1212859264
3		S wiggsiae 16S - primers	Undetermined	1212859264
3		S wiggsiae 16S - primers	Undetermined	1212859264
3		S wiggsiae 16S - primers	4.298	121285928
3		S wiggsiae 16S - primers	4.43	121285928
3		S wiggsiae 16S - primers	4.14	121285928
3		S wiggsiae 16S - primers	7.7	12128593
3		S wiggsiae 16S - primers	7.564	12128593
3		S wiggsiae 16S - primers	7.509	12128593
3		S wiggsiae 16S - primers	11.41	1212859.25
3		S wiggsiae 16S - primers	11.031	1212859.25
3		S wiggsiae 16S - primers	11.57	1212859.25
3		S wiggsiae 16S - primers	15.507	121285.93
3		S wiggsiae 16S - primers	15.272	121285.93
3		S wiggsiae 16S - primers	15.801	121285.93
3		S wiggsiae 16S - primers	18.431	12128.593
3		S wiggsiae 16S - primers	18.413	12128.593
3		S wiggsiae 16S - primers	18.681	12128.593
3		S wiggsiae 16S - primers	22.144	1212.859
3		S wiggsiae 16S - primers	21.76	1212.859
3		S wiggsiae 16S - primers	22.069	1212.859
3		S wiggsiae 16S - primers	28.327	121.286
3		S wiggsiae 16S - primers	30.713	121.286
3		S wiggsiae 16S - primers	29.861	12.129
3		S wiggsiae 16S - primers	30.064	12.129
3		S wiggsiae 16S - primers	33.852	12.129
5	NTC	Fnucleatum	Undetermined	
5	NTC	Fnucleatum	Undetermined	
5	NTC	Fnucleatum	Undetermined	
5		Fnucleatum	4.099	11261404200
5		Fnucleatum	4.04	11261404200
5		Fnucleatum	4.052	11261404200
5		Fnucleatum	7.128	1126140420
5		Fnucleatum	6.959	1126140420
5		Fnucleatum	7.024	1126140420
5		Fnucleatum	11.448	112614048
5		Fnucleatum	11.326	112614048
5		Fnucleatum	11.44	112614048
5		Fnucleatum	16.199	11261405
5		Fnucleatum	15.934	11261405
5		Fnucleatum	15.969	11261405
5		Fnucleatum	18.443	1126140.5
5		Fnucleatum	18.37	1126140.5
5		Fnucleatum	18.595	1126140.5
5		Fnucleatum	21.853	112614.05
5		Fnucleatum	21.652	112614.05
5		Fnucleatum	21.743	112614.05
5		Fnucleatum	25.741	11261.404
5		Fnucleatum	25.729	11261.404
5		Fnucleatum	25.88	11261.404
5		Fnucleatum	29.763	1126.1405
5		Fnucleatum	29.617	1126.1405

5		Fnucleatum	29.722	1126.1405
5		Fnucleatum	33.804	112.614044
5		Fnucleatum	33.793	112.614044
5		Fnucleatum	34.928	112.614044
5		Fnucleatum	36.247	11.261405
5		Fnucleatum	37.777	11.261405
5		Fnucleatum	39.19	11.261405
5		Fnucleatum	Undetermined	1.1261405
5		Fnucleatum	Undetermined	1.1261405
5		Fnucleatum	37.786	1.1261405
5	A19	Fnucleatum	29.875	1011.042
5	A19	Fnucleatum	30.035	914.89764
5	A19	Fnucleatum	30.05	906.3587
5	B19	Fnucleatum	Undetermined	
5	B19	Fnucleatum	Undetermined	
5	B19	Fnucleatum	Undetermined	
5	C19	Fnucleatum	Undetermined	
5	C19	Fnucleatum	Undetermined	
5	C19	Fnucleatum	Undetermined	
5		Swiggsae	Undetermined	
5		Swiggsae	Undetermined	
5		Swiggsae	Undetermined	
5		Swiggsae	3.963	12128592900
5		Swiggsae	Undetermined	12128592900
5		Swiggsae	Undetermined	12128592900
5		Swiggsae	7.283	1212859260
5		Swiggsae	7.169	1212859260
5		Swiggsae	7.208	1212859260
5		Swiggsae	12.388	121285928
5		Swiggsae	12.281	121285928
5		Swiggsae	12.592	121285928
5		Swiggsae	16.369	12128593
5		Swiggsae	15.964	12128593
5		Swiggsae	15.64	12128593
5		Swiggsae	19.97	1212859.2
5		Swiggsae	19.31	1212859.2
5		Swiggsae	19.66	1212859.2
5		Swiggsae	23.346	121285.93
5		Swiggsae	23.035	121285.93
5		Swiggsae	24.326	121285.93
5		Swiggsae	25.405	12128.593
5		Swiggsae	25.534	12128.593
5		Swiggsae	25.376	12128.593
5		Swiggsae	29.835	1212.8593
5		Swiggsae	30.145	1212.8593
5		Swiggsae	30.34	1212.8593
5		Swiggsae	33.901	121.28593
5		Swiggsae	33.886	121.28593
5		Swiggsae	33.735	121.28593
5		Swiggsae	37.983	12.128593
5		Swiggsae	36.713	12.128593
5		Swiggsae	37.897	12.128593
5		Swiggsae	Undetermined	1.2128593
5		Swiggsae	36.998	1.2128593
5		Swiggsae	40.427	1.2128593
5	A19	Swiggsae	39.713	2.1240828
5	A19	Swiggsae	38.418	4.887615
5	A19	Swiggsae	38.71	4.050023
5	B19	Swiggsae	Undetermined	
5	B19	Swiggsae	Undetermined	
5	B19	Swiggsae	Undetermined	
5	C19	Swiggsae	Undetermined	
5	C19	Swiggsae	40.195	1.5574207
5	C19	Swiggsae	Undetermined	
6	NTC	S mutans UA159	Undetermined	
6	NTC	S mutans UA159	Undetermined	
6	NTC	S mutans UA159	Undetermined	
6		S mutans UA159	4.387	18903502800
6		S mutans UA159	4.358	18903502800
6		S mutans UA159	4.412	18903502800
6		S mutans UA159	7.005	1890350210
6		S mutans UA159	6.963	1890350210
6		S mutans UA159	6.99	1890350210
6		S mutans UA159	10.017	189035024
6		S mutans UA159	10.044	189035024
6		S mutans UA159	9.994	189035024

6		S mutans UA159	13.575	18903502
6		S mutans UA159	13.516	18903502
6		S mutans UA159	13.587	18903502
6		S mutans UA159	17.289	1890350.2
6		S mutans UA159	17.257	1890350.2
6		S mutans UA159	17.177	1890350.2
6		S mutans UA159	20.443	189035.02
6		S mutans UA159	20.442	189035.02
6		S mutans UA159	20.608	189035.02
6		S mutans UA159	24.47	18903.502
6		S mutans UA159	24.411	18903.502
6		S mutans UA159	24.552	18903.502
6		S mutans UA159	27.184	1890.3502
6		S mutans UA159	27.332	1890.3502
6		S mutans UA159	27.317	1890.3502
6		S mutans UA159	31.617	189.03502
6		S mutans UA159	31.506	189.03502
6		S mutans UA159	31.61	189.03502
6		S mutans UA159	34.889	18.903502
6		S mutans UA159	34.952	18.903502
6		S mutans UA159	35.964	18.903502
6		S mutans UA159	38.402	1.8903502
6		S mutans UA159	Undetermined	1.8903502
6		S mutans UA159	Undetermined	1.8903502
6	A19	S mutans UA159	Undetermined	
6	A19	S mutans UA159	Undetermined	
6	A19	S mutans UA159	38.988	1.1470077
6	B19	S mutans UA159	Undetermined	
6	B19	S mutans UA159	Undetermined	
6	B19	S mutans UA159	Undetermined	
6	C19	S mutans UA159	Undetermined	
6	C19	S mutans UA159	Undetermined	
6	C19	S mutans UA159	Undetermined	
6	DX FTA	S mutans UA159	Undetermined	
6	DX FTA	S mutans UA159	Undetermined	
6	DX FTA	S mutans UA159	Undetermined	
6	DX swab	S mutans UA159	38.016	2.1850295
6	DX swab	S mutans UA159	36.947	4.4395914
6	DX swab	S mutans UA159	37.193	3.770965
7		R dentocariosa R aeria 16S	36.734	
7		R dentocariosa R aeria 16S	36.622	
7		R dentocariosa R aeria 16S	38.742	
7		R dentocariosa R aeria 16S	4.321	17428076500
7		R dentocariosa R aeria 16S	4.233	17428076500
7		R dentocariosa R aeria 16S	4.397	17428076500
7		R dentocariosa R aeria 16S	7.042	1742807810
7		R dentocariosa R aeria 16S	6.865	1742807810
7		R dentocariosa R aeria 16S	7.015	1742807810
7		R dentocariosa R aeria 16S	10.94	174280768
7		R dentocariosa R aeria 16S	10.743	174280768
7		R dentocariosa R aeria 16S	11.014	174280768
7		R dentocariosa R aeria 16S	14.202	17428078
7		R dentocariosa R aeria 16S	14.259	17428078
7		R dentocariosa R aeria 16S	14.292	17428078
7		R dentocariosa R aeria 16S	20.588	1742807.8
7		R dentocariosa R aeria 16S	21.556	1742807.8
7		R dentocariosa R aeria 16S	22.326	1742807.8
7		R dentocariosa R aeria 16S	20.252	174280.78
7		R dentocariosa R aeria 16S	22.328	174280.78
7		R dentocariosa R aeria 16S	22.614	174280.78
7		R dentocariosa R aeria 16S	Undetermined	17428.078
7		R dentocariosa R aeria 16S	23.868	17428.078
7		R dentocariosa R aeria 16S	24.971	17428.078
7		R dentocariosa R aeria 16S	24.214	1742.8077
7		R dentocariosa R aeria 16S	23.98	1742.8077
7		R dentocariosa R aeria 16S	24.232	1742.8077
7		R dentocariosa R aeria 16S	34.429	174.28078
7		R dentocariosa R aeria 16S	34.201	174.28078
7		R dentocariosa R aeria 16S	34.791	174.28078
7		R dentocariosa R aeria 16S	37.448	17.428078
7		R dentocariosa R aeria 16S	39.754	17.428078
7		R dentocariosa R aeria 16S	36.241	17.428078
7		R dentocariosa R aeria 16S	36.626	1.7428077
7		R dentocariosa R aeria 16S	Undetermined	1.7428077
7		R dentocariosa R aeria 16S	37.852	1.7428077
7	A19	R dentocariosa R aeria 16S	31.401	293.80182

7	A19	R dentocariosa R aeria 16S	32.427	149.57208
7	A19	R dentocariosa R aeria 16S	32.535	139.32016
7	B19	R dentocariosa R aeria 16S	28.139	2513.058
7	B19	R dentocariosa R aeria 16S	28.064	2639.6921
7	B19	R dentocariosa R aeria 16S	28.249	2337.1482
7	C19	R dentocariosa R aeria 16S	26.764	6210.293
7	C19	R dentocariosa R aeria 16S	26.527	7259.62
7	C19	R dentocariosa R aeria 16S	26.843	5896.634
7	DX FTA	R dentocariosa R aeria 16S	26.592	6953.325
7	DX FTA	R dentocariosa R aeria 16S	26.411	7835.1216
7	DX FTA	R dentocariosa R aeria 16S	26.621	6822.7563
7	DX swab	R dentocariosa R aeria 16S	22.614	95266.99
7	DX swab	R dentocariosa R aeria 16S	22.276	119062.51
7	DX swab	R dentocariosa R aeria 16S	21.972	145387.89
Pging rerun	NTC	P gingivalis spp hmuY	Undetermined	
Pging rerun	NTC	P gingivalis spp hmuY	Undetermined	
Pging rerun	NTC	P gingivalis spp hmuY	Undetermined	
Pging rerun	NTC	P gingivalis spp hmuY	Undetermined	
Pging rerun	NTC	P gingivalis spp hmuY	Undetermined	
Pging rerun	NTC	P gingivalis spp hmuY	Undetermined	
Pging rerun		P gingivalis spp hmuY	5.097	1000000000.00
Pging rerun		P gingivalis spp hmuY	4.871	1000000000.00
Pging rerun		P gingivalis spp hmuY	5.011	1000000000.00
Pging rerun		P gingivalis spp hmuY	8.714	100000000.00
Pging rerun		P gingivalis spp hmuY	7.981	100000000.00
Pging rerun		P gingivalis spp hmuY	8.308	100000000.00
Pging rerun		P gingivalis spp hmuY	11.771	10000000.00
Pging rerun		P gingivalis spp hmuY	11.74	10000000.00
Pging rerun		P gingivalis spp hmuY	11.905	10000000.00
Pging rerun		P gingivalis spp hmuY	15.046	1000000.00
Pging rerun		P gingivalis spp hmuY	14.76	1000000.00
Pging rerun		P gingivalis spp hmuY	15.055	1000000.00
Pging rerun		P gingivalis spp hmuY	18.373	100000.00
Pging rerun		P gingivalis spp hmuY	18.147	100000.00
Pging rerun		P gingivalis spp hmuY	18.602	100000.00
Pging rerun		P gingivalis spp hmuY	21.726	10000.00
Pging rerun		P gingivalis spp hmuY	21.626	10000.00
Pging rerun		P gingivalis spp hmuY	21.831	10000.00
Pging rerun		P gingivalis spp hmuY	24.667	1000.00
Pging rerun		P gingivalis spp hmuY	24.117	1000.00
Pging rerun		P gingivalis spp hmuY	24.819	1000.00
Pging rerun		P gingivalis spp hmuY	26.989	100.00
Pging rerun		P gingivalis spp hmuY	25.99	100.00
Pging rerun		P gingivalis spp hmuY	26.644	100.00
Pging rerun	Sample 1 - A19	P gingivalis spp hmuY	34.978	0.41
Pging rerun	Sample 1 - A19	P gingivalis spp hmuY	36.24	0.16
Pging rerun	Sample 1 - A19	P gingivalis spp hmuY	36.708	0.12
Pging rerun	Sample 2 - B19	P gingivalis spp hmuY	Undetermined	
Pging rerun	Sample 2 - B19	P gingivalis spp hmuY	Undetermined	
Pging rerun	Sample 2 - B19	P gingivalis spp hmuY	Undetermined	
Pging rerun	Sample 3 - C19	P gingivalis spp hmuY	37.807	0.05
Pging rerun	Sample 3 - C19	P gingivalis spp hmuY	Undetermined	
Pging rerun	Sample 3 - C19	P gingivalis spp hmuY	Undetermined	

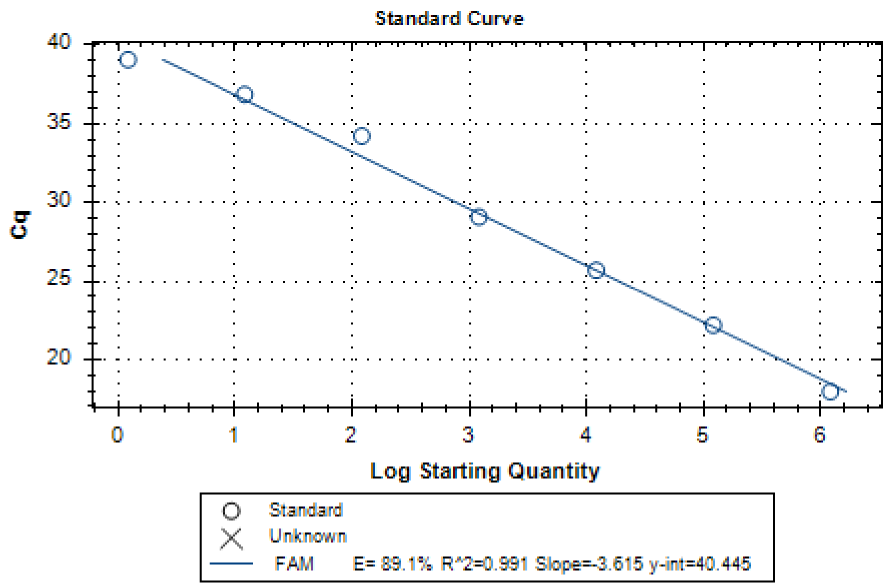

Supplement 3 Figure 1 Standard curve for S. wiggsiae at optimal annealing temperature of 63 °C run on CFX-384 (Bio-Rad).

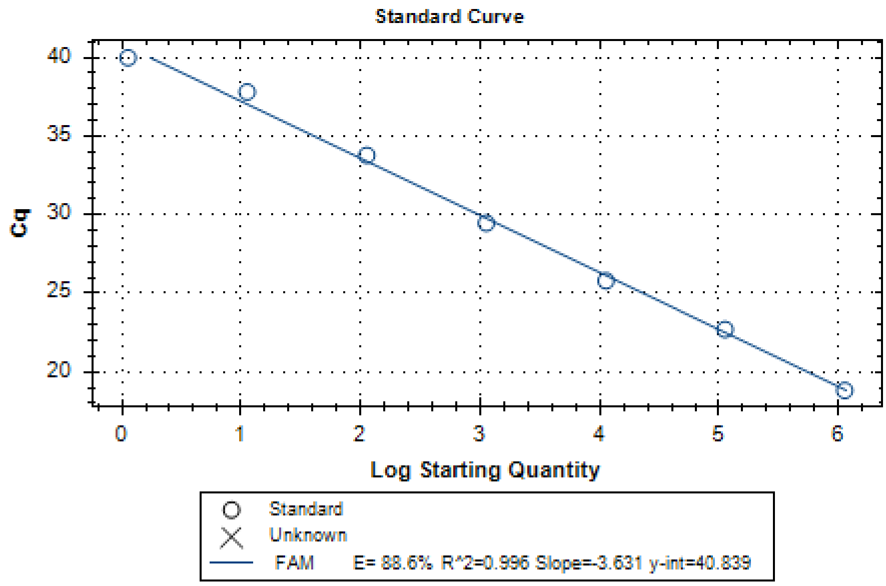

Supplement 3 Figure 2 Standard curve for F. nucleatum subspp. fusiforme at optimal annealing temperature of 60.7 °C run on CFX-384 (Bio-Rad).

Supplement 4

Tapestation analysis on D1000 Screentape

First sample (A1) *S. mutans* sample
Well number J3 (technical replicates are J4 and J5, expected amplicon length and size of expected band= 218bp; gBlocks length = 258bp)

Second sample(B1) *S. wiggsiae* sample
-Well number J7 (technical replicates in J8, J9, expected amplicon length and size of expected band = 161bp, gBlocks length =201bp)

Background:The *S. mutans* and *S. wiggsiae* assays showed no amplification, so the qPCR products from the mid-point dilutions of gBlocks standards (18903.5 and 12128.6 gene copies/ µL respectively) were run on gel electrophoresis (Tapestation).
Interpretation: the results suggested that no products were formed from the qPCR reaction.

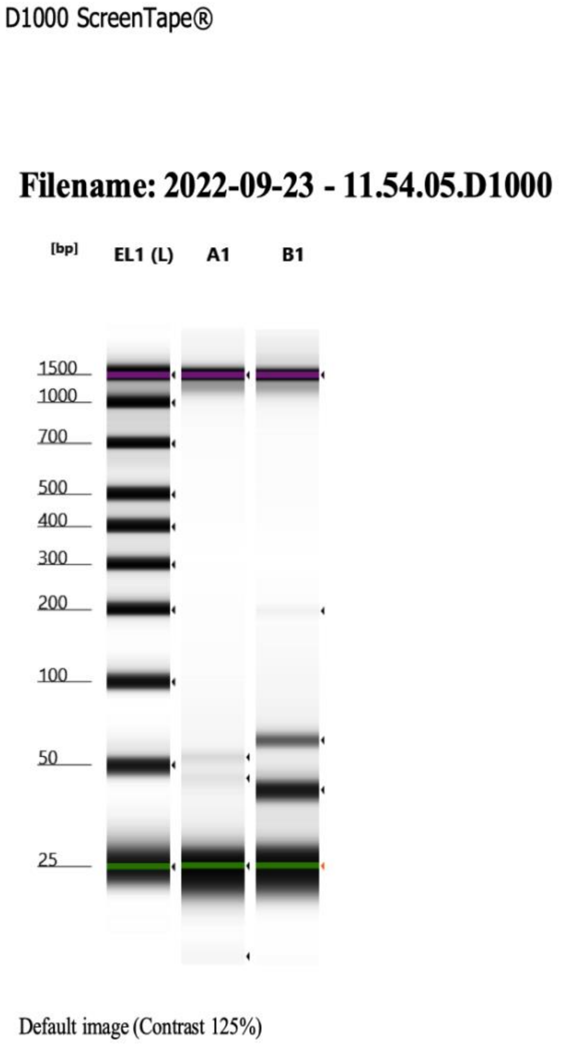

Supplement 5

Sanger sequencing and validation of qPCR assay products

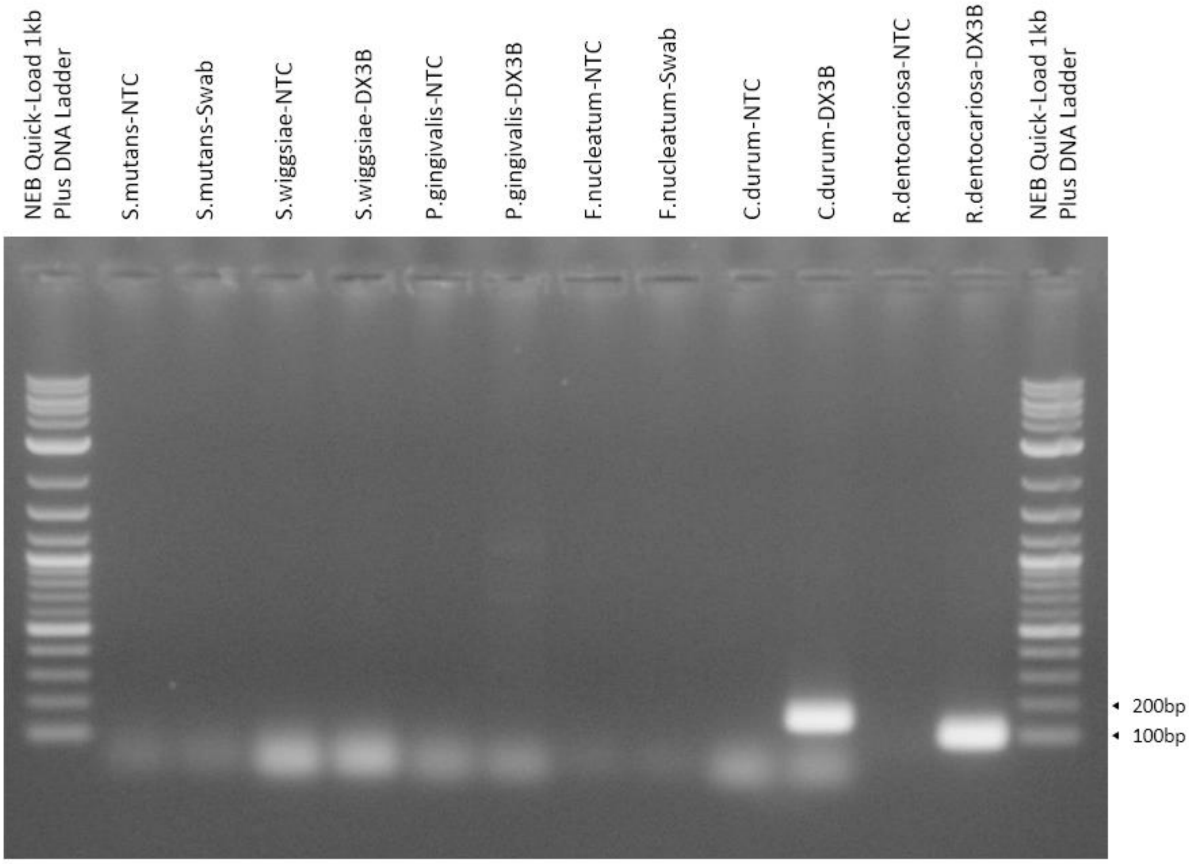

Figure 1a. Standard PCR performed on non-precious samples taken from FTA cards.

Performed at 55°C annealing temperature on 13 September 2023. Non-precious samples were taken from participants who consented to swabbing for validation purposes. Results showed a single band for C.durum at ~150bp and for R.dentocariosa at ~100bp respectively. No bands were visible on the bacterial other species S. mutans, S. wiggsiae, P. gingivalis and F. nucleatum subspp. fusiforme. NEB= New England Biolabs; NTC= no template control

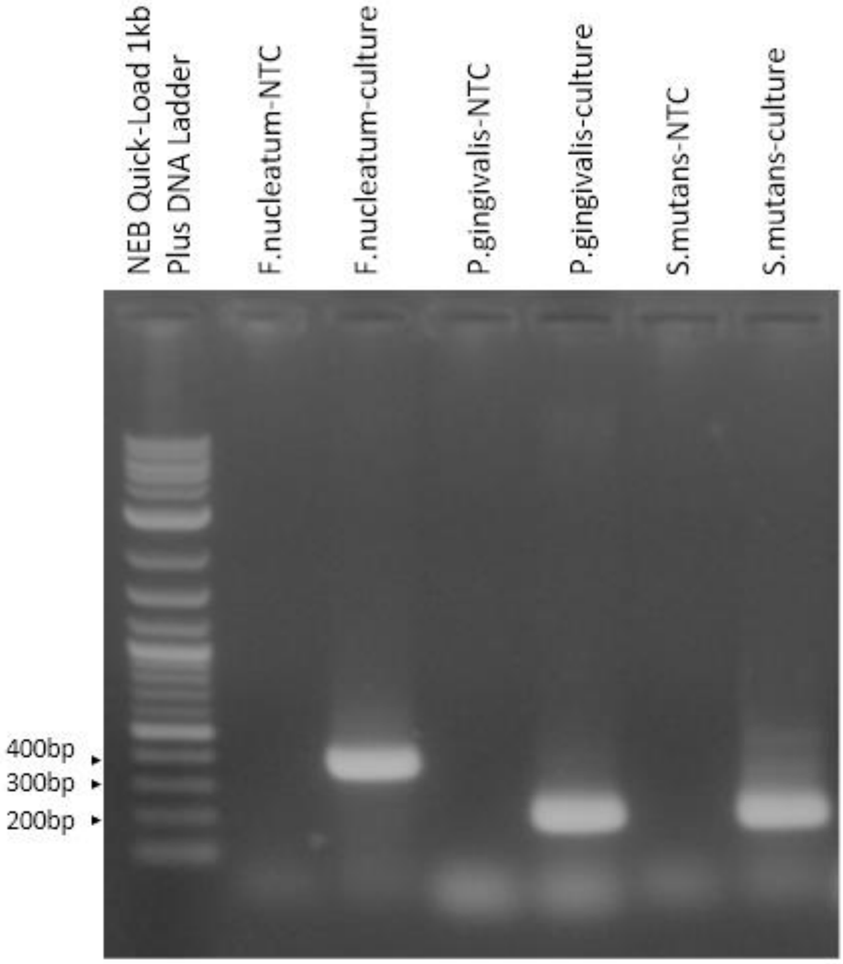

Figure 1b
Performed at 55°C annealing temperature on 3 October 2023. Non-precious samples were taken from broth culture which contained the respective species F nucleatum subspp. fusiforme, P gingivalis and S. mutans. Results showed a single band for S. mutans, S. wiggsiae, P. gingivalis and F. nucleatum subspp. fusiforme.
NEB= New England Biolabs; NTC= no template control

Table 1. Multiple sequence alignment results for C durum target amplicon and Sanger sequencing Forward reading

```
CLUSTAL W (1.83) multiple sequence alignment

Amplicon        GGCTGTGTGTTTGCAGTCTGTGCCGTAGCTAACGCATTAAGCGCCCCGCC
Sanger          TGCCA-------------------------------TT-AGCGC-CCGCC
                 **                                 ** ***** *****

Amplicon        TGGGGAGTACGGCCGCAAGGCTAAAACTCAAAGGAATTGACGGGGGCCCG
Sanger          TGGGGAGTACGGCCGCAAGGCTA-AACTCAAAGGAATTGACGGGGGCCCG
                *********************** **************************

Amplicon        CACAAGCGGCGGAGCATGTGGATTAATTCGATGCAACGCGAAGAACCT--
Sanger          CACAAGCGGCGGAGCATGTGGATTAATTCGATGCAACGCGAAGAACCTAT
                ************************************************

Amplicon        TA
Sanger          AA
                 *
```

Legend: Blue highlight denotes an aligned sequence, with exception of single nucleotide polymorphism (shown in yellow highlight)

```
1      GGCTGTGTGT  TTGCAGTCTG  TGCCGTAGCT  AACGCATTAA  GCGCCCCGCC
51     TGGGGAGTAC  GGCCGCAAGG  CTAAAACTCA  AAGGAATTGA  CGGGGGCCCG
101    CACAAGCGGC  GGAGCATGTG  GATTAATTCG  ATGCAACGCG  AAGAACCTTA
```

Figure 2. MSA For C Durum Sanger forward reading sequencing results (including SNPs) superimposed on Primer3Plus output of amplicon.

The entire target amplicon sequence for C durum is shown, with forward primer (purple), probe (black), reverse primer (yellow) and multiple sequence alignment result for the amplicon and the Sanger sequencing product (blue highlight)

Table 2. Multiple sequence alignment results for C durum target amplicon and Sanger sequencing reverse complement of the reverse reading

```
CLUSTAL W (1.83) multiple sequence alignment

Amplicon_Cd                      GG--CTGTGTGTTTGCAGTCTGTGCCGTAGCTAACGCATTAAGCGCCCCG
Sanger_Cd_reverse_revcomplement  TGGGCTGTGTGTTTGCAGTCTGTGCCGTAGCTAACGCATTAAGCGCCCCG
                                  *  **********************************************

Amplicon_Cd                      CCTGGGGAGTACGGCCGCAAGGCTAAAACTCAAAGGAATTGACGGGGGCC
Sanger_Cd_reverse_revcomplement  CCTGGGGAGTACGGCCGCAAGGCTAAAACTCAAAGGAA-TGACGGGGGC-
                                 ************************************** **********

Amplicon_Cd                      CGCACAAGCGGCGGAGCATGTGGATTAATTCGATGCAACGCGAAGAACCT
Sanger_Cd_reverse_revcomplement  CGCACAAGCG------------------------------CGA---G---
                                 **********                              ***

Amplicon_Cd                      TA
Sanger_Cd_reverse_revcomplement  CG
```

Legend: Blue highlight denotes an aligned sequence, with exception of single nucleotide polymorphism (shown in yellow highlight)

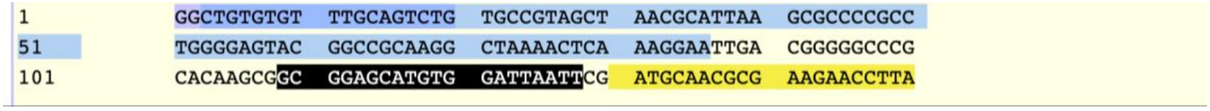

Figure 3. MSA For C Durum Sanger reverse complement for the reverse reading sequencing results (including SNPs) superimposed on Primer3Plus output of amplicon.

The entire target amplicon sequence for C durum is shown, with forward primer (purple), probe (black), reverse primer (yellow) and multiple sequence alignment result for the amplicon and the Sanger sequencing product (blue highlight)

Table 3. Multiple sequence alignment results for R. dentocariosa/R. aeria target amplicon and Sanger sequencing product (forward reading)

```
CLUSTAL W (1.83) multiple sequence alignment

Amplicon_Rd     CGTGAGTGACCTACCTTTGACTCTGGGATAAGCCTGGGAAACTGGGTCTA
Sanger_Rd       GC--------CTG--------------------------CACTGGGTCT-
                          **                            ********* 

Amplicon_Rd     ATACCGGATACGACCAATCTCCGCATGGGGTGTTGGTGGAAAGCGTTATG
Sanger_Rd       ATACCGGATACGACCAATCTCTGCATGGGGTGTTGGTGGAAAGCGTTATG
                ********************* ****************************
```

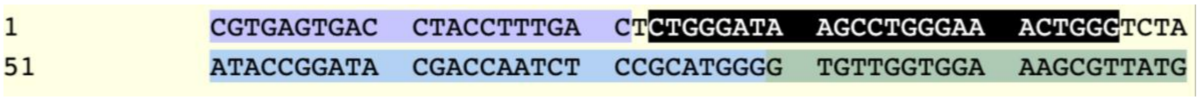

Figure 4. MSA For R. dentocariosa Sanger forward reading sequencing results superimposed on Primer3Plus output of amplicon.

The entire target amplicon sequence for C durum is shown, with forward primer (purple), probe (black), reverse primer (yellow) and multiple sequence alignment result for the amplicon and the Sanger sequencing product (blue highlight)

Table 4. Multiple sequence alignment results for R dentocariosa target amplicon and Sanger sequencing reverse complement of the reverse reading

```
CLUSTAL W (1.83) multiple sequence alignment

Rd_amplicon                      CG---TGA-GTGACCTACCTTTGACTCTGGGATAAGCCTGGGAAACTGGG
Rd_sanger_rev_reversecomplement  CTCTGTGACGTGACCTACCTTTGACTCTGGGATAAGCCTGGGAAACTGGG
                                 *    *** *****************************************

Rd_amplicon                      TCTAATACCGGATACGACCAATCTCCGCATGGGGTGTTGGTGGAAAGCGT
Rd_sanger_rev_reversecomplement  TCTAATACCG-ATACGAC--------------------------------
                                 ********** *******

Rd_amplicon                      TATG
Rd_sanger_rev_reversecomplement  -ATG
                                  ***
```

Legend: Blue highlight denotes an aligned sequence, with exception of single nucleotide polymorphism (shown in yellow highlight)

```
1      CGTGAGTGAC  CTACCTTTGA  CTCTGGGATA  AGCCTGGGAA  ACTGGGTCTA
51     ATACCGGATA  CGACCAATCT  CCGCATGGGG  TGTTGGTGGA  AAGCGTTATG
```

Figure 5. MSA For R dentocariosa Sanger reverse complement for the reverse reading sequencing results superimposed on Primer3Plus output of amplicon.

The entire target amplicon sequence for C durum is shown, with forward primer (purple), probe (black), reverse primer (yellow) and multiple sequence alignment result for the amplicon and the Sanger sequencing product (blue highlight)

Table 5. Multiple sequence alignment results for F. nucleatum subspp. fusiforme target amplicon and Sanger sequencing Forward reading

```
CLUSTAL W (1.83) multiple sequence alignment

Fn_amplicon         GATGAGGATGAAAAGAAACAAAGTATAGAGAACTCAAAAACAATTTTAAA
Fn_sanger_forward   AC-------------AA----------------------ACA-TTTT-AA
                                   **                      *** **** **

Fn_amplicon         TAATAGAGATATAGAAAATTTTATATCTAAAAATATGGCAAGTTATAACT
Fn_sanger_forward   TAATAGAGATATAGAA-ATTTTATATCTAA-AATATGGCAAGTTATAACT
                    **************** ************* *******************

Fn_amplicon         TTAAGAAAAAAGAAGTTATATCAGAATATAAACATTCTTCAGTAGAATGC
Fn_sanger_forward   TTAAGAAAAAAGAAGTTATATCAGAATATAAACATTCTTCAGTAGAATGC
                    **************************************************

Fn_amplicon         TTCTCTTATGAATTAAAGAAAGAAGTGGCAATTGATATAGACAAAGAAAA
Fn_sanger_forward   TTCTCTTATGAATTAAAGAAAGAAGTGGCAATTGATATAGACAAAGAAAA
                    **************************************************

Fn_amplicon         ATTATCTTTAATAGTAAAAAATAAAATGAGAGATTTGTATATAAAAGCTA
Fn_sanger_forward   ATTATCTTTAATAGTAAAAAATAAAATGAGAGATTTGTATATAAAAGCTA
                    **************************************************

Fn_amplicon         ATTATTTTGTAGATGATTTGTCAAAGGTTATAGCTAAAGAAAAACAGTAT
Fn_sanger_forward   ATTATTTTGTAGATGATTTGTCAAAGGTTATAGCTAAAGAAAAACAGTAT
                    **************************************************

Fn_amplicon         AGATTCTCTGATAATGATGTATCAGAAAATCTTAAAGACTATGAATATTC
Fn_sanger_forward   AGATTCTCTGATAATGATGTATCAGAAAATCTTAAAGACTATGAATATTC
                    **************************************************

Fn_amplicon         AGATATAAAGAATATAGTAAAAGTCAATAGCCCTTCT-C--AATGG
Fn_sanger_forward   AGATATAAAGAATATAGTAAAAGTCAATAGTTTTGCTTCAAATGGG
                    ******************************   * ** *  *  **
```

Legend: Blue highlight denotes an aligned sequence, with exception of single nucleotide polymorphism (shown in yellow highlight)

1	GATGAGGATG	AAAAGAAACA	AAGTATAGAG	AACTCAAAAA	CAATTTTAAA
51	TAATAGAGAT	ATAGAAAATT	TTATATCTAA	AAATATGGCA	AGTTATAACT
101	TTAAGAAAAA	AGAAGTTATA	TCAGAATATA	AACATTCTTC	AGTAGAATGC
151	TTCTCTTATG	AATTAAAGAA	AGAAGTGGCA	ATTGATATAG	ACAAAGAAAA
201	ATTATCTTTA	ATAGTAAAAA	ATAAAATGAG	AGATTTGTAT	ATAAAAGCTA
251	ATTATTTTGT	AGATGATTTG	TCAAAGGTTA	TAGCTAAAGA	AAAACAGTAT
301	AGATTCTCTG	ATAATGATGT	ATCAGAAAAT	CTTAAAGACT	ATGAATATTC
351	AGATATAAAG	AATATAGTAA	AAGTCAATAG	CCCTTCTCAA	TGG

Figure 6. MSA for F. nucleatum subspp. fusiforme Sanger forward reading sequencing results (including SNPs) superimposed on Primer3Plus output of amplicon.

The entire target amplicon sequence for F. nucleatum subspp. fusiforme is shown, with forward primer (purple), probe (black), reverse primer (yellow) and multiple sequence alignment result for the amplicon and the Sanger sequencing product (blue highlight)

Table 6. Multiple sequence alignment results for F. nucleatum subspp. fusiforme target amplicon and Sanger sequencing reverse complement of the reverse reading

```
CLUSTAL W (1.83) multiple sequence alignment

Fn_amplicon                     GATGAGGATGAAAAGAAACAAAGTATAGAGAACTCAAAAACAATTTTAAA
Fn_sanger_rev_revcomplement     AA------------------------------------AACAATTTTAAA
                                 *                                    ************

Fn_amplicon                     TAATAGAGATATAGAAAATTTTATATCTAAAAATATGGCAAGTTATAACT
Fn_sanger_rev_revcomplement     TAATAGAGATATAGAAAATTTTATATCTAAAAATATGGCAAGTTATAACT
                                **************************************************

Fn_amplicon                     TTAAGAAAAAAGAAGTTATATCAGAATATAAACATTCTTCAGTAGAATGC
Fn_sanger_rev_revcomplement     TTAAGAAAAAAGAAGTTATATCAGAATATAAACATTCTTCAGTAGAATGC
                                **************************************************

Fn_amplicon                     TTCTCTTATGAATTAAAGAAAGAAGTGGCAATTGATATAGACAAAGAAAA
Fn_sanger_rev_revcomplement     TTCTCTTATGAATTAAAGAAAGAAGTGGCAATTGATATAGACAAAGAAAA
                                **************************************************

Fn_amplicon                     ATTATCTTTAATAGTAAAAAATAAAATGAGAGATTTGTATATAAAAGCTA
Fn_sanger_rev_revcomplement     ATTATCTTTAATAGTAAAAAATAAAATGAGAGATTTGTATATAAAAGCTA
                                **************************************************

Fn_amplicon                     ATTATTTTGTAGATGATTTGTCAAAGGTTATAGCTAAAGAAAAAC-AGTA
Fn_sanger_rev_revcomplement     ATTATTTTGTAGATGATTTGTCAAAGGTTATAGCTAAAGAAAAACTAGTA
                                ********************************************* ****

Fn_amplicon                     TAGATTCTCTGATAATGATGTATCAGAAAATCTTAAAGACTATGAATATT
Fn_sanger_rev_revcomplement     TAGATTCTCTGATAATGATGTATCAGAAAATCT-AAAGACTATGAATATC
                                ********************************* *************** 

Fn_amplicon                     CAGATATAAAGAATATAGTAAAAGTCAATAGCCCTTCTCAATGG
Fn_sanger_rev_revcomplement     CAGAT-------------------------------------AG
                                *****                                     *
```

Legend: Blue highlight denotes an aligned sequence, with exception of single nucleotide polymorphism (shown in yellow highlight)

```
1      GATGAGGATG  AAAAGAAACA  AAGTATAGAG  AACTCAAAAA  CAATTTTAAA
51     TAATAGAGAT  ATAGAAAATT  TTATATCTAA  AAATATGGCA  AGTTATAACT
101    TTAAGAAAAA  AGAAGTTATA  TCAGAATATA  AACATTCTTC  AGTAGAATGC
151    TTCTCTTATG  AATTAAAGAA  AGAAGTGGCA  ATTGATATAG  ACAAAGAAAA
201    ATTATCTTTA  ATAGTAAAAA  ATAAAATGAG  AGATTTGTAT  ATAAAAGCTA
251    ATTATTTTGT  AGATGATTTG  TCAAAGGTTA  TAGCTAAAGA  AAAACAGTAT
301    AGATTCTCTG  ATAATGATGT  ATCAGAAAAT  CTTAAAGACT  ATGAATATTC
351    AGATATAAAG  AATATAGTAA  AAGTCAATAG  CCCTTCTCAA  TGG
```

Figure 7. MSA For F. nucleatum subspp. fusiforme Sanger reverse complement for the reverse reading sequencing results superimposed on Primer3Plus output of amplicon.

The entire target amplicon sequence for F. nucleatum subspp. fusiforme is shown, with forward primer (purple), probe (black), reverse primer (yellow) and multiple sequence alignment result for the amplicon and the Sanger sequencing product (blue highlight)

Table 7. Multiple sequence alignment results for P. gingivalis- target amplicon and Sanger sequencing Forward reading

```
Pg_amplicon         GCTTCGAAATACGAAACGTGGCAGTATTTCTCTTTTTCCAAAGGTGAAGT
Pg_sanger_forward   CCT---GAATAC------------------------------GGTG-AGT
                     **    *****                              **** ***

Pg_amplicon         CGTAAATGTTACCGACTATAAGAACGATTTGAACTGGGACATGGCTCTTC
Pg_sanger_forward   CGTA-ATGTTACCGACTATAAGAACGATTTGAACTGGGACATGGCTCTTC
                    **** *********************************************

Pg_amplicon         ACCGCTATGACGTTCGTCTCAATTGTGGCGAAAGTGGCAAGGGAAAAGGT
Pg_sanger_forward   ACCGCTATGACGTTCGTCTCAATTGTGGCGAAAGTGGTAAGGGAAAAGGT
                    ************************************* ************

Pg_amplicon         GGTGCCGTATTCTCCGGCAAGACAGAAATGGATCAGGCTACTTCCGTTCC
Pg_sanger_forward   GGTGCCGTATTCTCCGGCAAGACAGAAATGGATCAGGCTACTACCGTTCC
                    ****************************************** *******

Pg_amplicon         GACAGACGGATA--------------------------------------
Pg_sanger_forward   GACAGACGGATATAAAAATATATGAGAGATTTGTATATGAAAGCTAATTA
                    ************

Pg_amplicon         --------------------------------------------------
Pg_sanger_forward   TTTTGTAGATGATTTGTCAAAGGTTATAGCTAAAGAAAAACAGTATAAAT

Pg_amplicon         --------------------------------------------------
Pg_sanger_forward   TCTCTGATAATGATGTATCAGAAAATCTTAAAGACTATGAATATTCAGAT

Pg_amplicon         ----------------------------------------TA
Pg_sanger_forward   ATAAAGAGATAGTAAAAGTCAATAGCTTTTGTTAAATCGGAC
```

Legend: Blue highlight denotes an aligned sequence, with exception of single nucleotide polymorphism (shown in yellow highlight)

1	GCTTCGAAAT	ACGAAACGTG	GCAGTATTTC	TCTTTTTCCA	AAGGTGAAGT
51	CGTAAATGTT	ACCGACTATA	AGAACGATTT	GAACTGGGAC	ATGGCTCTTC
101	ACCGCTATGA	CGTTCGTCTC	AATTGTGGCG	AAAGTGGCAA	GGGAAAAGGT
151	GGTGCCGTAT	TCTCCGGCAA	GACAGAAATG	GATCAGGCTA	CTTCCGTTCC
201	GACAGACGGA	TATA			

Figure 8. MSA for P.gingivalis Sanger forward reading sequencing results (including SNPs) superimposed on Primer3Plus output of amplicon.

The entire target amplicon sequence for P. gingivalis is shown, with forward primer (purple), probe (black), reverse primer (yellow) and multiple sequence alignment result for the amplicon and the Sanger sequencing product (blue highlight)

Table 8. Multiple sequence alignment results for P. gingivalis target amplicon and Sanger sequencing reverse complement of the reverse reading

```
CLUSTAL W (1.83) multiple sequence alignment

Pg_amplicon                       GCT-----------------------------------------------
Pg_sanger_rev_reversecomplement   TATTTTAATTAATAGAGATATAGAAATTGTGGTATCTCAAAATCAGGCAA
                                    *

Pg_amplicon                       --------------------------------------------------
Pg_sanger_rev_reversecomplement   GTTATAACCTTACGCAAAAAGAAGTTATATCGGAATATAATCATTCTTCA

Pg_amplicon                       -----------------------------------------TCGAAATAC
Pg_sanger_rev_reversecomplement   GTAGAATGCTTCTCTTCCGATTTCAAGAAAGAAGTGTTGCTTCGAAATAC
                                                                           *********

Pg_amplicon                       GAAACGTGGCAGTATTTCTCTTTTTCCAAAGGTGAAGTCGTAAATGTTAC
Pg_sanger_rev_reversecomplement   GAAACGTGGCAGTATTTCTCTTTTTCCAAAGGTGAAGTCGTAAATGTTAC
                                  **************************************************

Pg_amplicon                       CGACTATAAGAACGATTTGAACTGGGACATGGCTCTTCACCGCTATGACG
Pg_sanger_rev_reversecomplement   CGACTATAAGAACGATTTGAACTGGGACATGGCTCTTCACCGCTATGACG
                                  **************************************************

Pg_amplicon                       TTCGTCTCAATTGTGGCGAAAGTGGCAAGGGAAAAGGTGGTGCCGTATTC
Pg_sanger_rev_reversecomplement   TTCGTCTCAATTGTGGCGAAAGTGGTAAGGGAAAAGGTGGTGCCGTAT-C
                                  ************************* ********************** *

Pg_amplicon                       TCCGGCAAGACAGAAATGGATCAGGCTACTTCCGTTCCGACAGACGGATA
Pg_sanger_rev_reversecomplement   TCCGC--AGACAGAA-T------------------------------AT-
                                  ****   ******** *                             **

Pg_amplicon                       TA
Pg_sanger_rev_reversecomplement   AT
```

Legend: Blue highlight denotes an aligned sequence, with exception of single nucleotide polymorphism (shown in yellow highlight)

1	GCTTCGAAAT	ACGAAACGTG	GCAGTATTTC	TCTTTTTCCA	AAGGTGAAGT
51	CGTAAATGTT	ACCGACTATA	AGAACGATTT	GAACTGGGAC	ATGGCTCTTC
101	ACCGCTATGA	CGTTCGTCTC	AATTGTGGCG	AAAGTGGCAA	GGGAAAAGGT
151	GGTGCCGTAT	TCTCCGGCAA	GACAGAAATG	GATCAGGCTA	CTTCCGTTCC
201	GACAGACGGA	TATA			

Figure 17. MSA For P.gingivalis Sanger reverse complement for the reverse reading sequencing results superimposed on Primer3Plus output of amplicon.

The entire target amplicon sequence for P. gingivalis is shown, with forward primer (purple), probe (black), reverse primer (yellow) and multiple sequence alignment result for the amplicon and the Sanger sequencing product (blue highlight)

Table 9. Multiple sequence alignment results for S. mutans- target amplicon and Sanger sequencing Forward reading

```
CLUSTAL W (1.83) multiple sequence alignment

Smutans_amplicon         TGACTTGCTCCAAATTGCTGGGGATTACCTCAAAGCTGCTAAGGGGATCC
Smutans_sanger_forward   TA------------CTG---------------------CTACG--GA-CC
                         *              **                     *** *  ** **

Smutans_amplicon         ATAAAAATGATAAGGCTGCTAATGATCATTTGTCTATTTTAGAGGCATGG
Smutans_sanger_forward   ATAA-AATGATAAGGCTGCTAATGATCATTTGTCTATTTTAGAGGCATGG
                         **** *********************************************

Smutans_amplicon         AGTGACAACGACACTCCTTACCTTCATGATGATGGCGACAATATGATTAA
Smutans_sanger_forward   AGTTATAACGATACTCCTTACCTTCATGATGATGGCGACAATATGATTAA
                         *** * ***** **************************************

Smutans_amplicon         TATGGACAATAAGCTGCGTTTGTCTCTATTATTTTCATTAGCTAAACCCT
Smutans_sanger_forward   TATGGACAATAGGCTGCGTTTTTCCTTGCTTTATTCATTAGCTAAACCCT
                         *********** ********* **  *  * * *****************

Smutans_amplicon         TAAATCAACGTTCAGG----------------------------------
Smutans_sanger_forward   TAAATCAACGTTCAGGCAAAAAGAATCGTGAGATTTGTATAGAAAAGCTA
                         ****************

Smutans_amplicon         --------------------------------------------------
Smutans_sanger_forward   ATTAGTTTGTAGATGATTTTCCGAAGGTTCTACCTAACGAAAAACATTAT

Smutans_amplicon         --------------------------------------------------
Smutans_sanger_forward   AAATTCCCTGATAACGATGTATCACAAAAACTATAAGACTATGAATATAC

Smutans_amplicon         -------------------------------------------------C
Smutans_sanger_forward   TTATATGGAGACTAGAGTAAAAGTCAGTTGCTTTATGTTAAATGATGATT

Smutans_amplicon         A
Smutans_sanger_forward   T
```

Legend: Blue highlight denotes an aligned sequence, with exception of single nucleotide polymorphism (shown in yellow highlight)

1	tgacttgctc	caaattgctg	gggattacct	caaagctgct	aaggggatcc
51	ataaaaatga	taaggctgct	aatgatcatt	tgtctatttt	agaggcatgg
101	agtgacaacg	acactcctta	ccttcatgat	gatggcgaca	atatgattaa
151	tatggacaat	aagctgcgtt	tgtctctatt	attttcatta	gctaaaccct
201	taaatcaacg	ttcaggca			

Figure 19. MSA for S. mutans Sanger forward reading sequencing results (including SNPs) superimposed on Primer3Plus output of amplicon.

The entire target amplicon sequence for S. mutans is shown, with forward primer (purple), probe (black), reverse primer (yellow) and multiple sequence alignment result for the amplicon and the Sanger sequencing product (blue highlight)

Table 10. Multiple sequence alignment results for S. mutans target amplicon and Sanger sequencing reverse complement of the reverse reading

```
CLUSTAL W (1.83) multiple sequence alignment

Smutans_amplicon            TG------------------------------------------------
Smutans_sanger_rev_revcomp  TTATGCCGTCCCTCTACAAGGAGCACTTTCGGCATATCATCTCCCTTCTG
                            *

Smutans_amplicon            -----------------------------------ACTTGCTCCAAATTG
Smutans_sanger_rev_revcomp  TTTATCTTTTTTTTTCACATCTTTCGTATGTCTTGACTTGCTCCAAATTG
                                                               ***************

Smutans_amplicon            CTGGGGATTACCTCAAAGCTGCTAAGGGGATCCATAAAAATGATAAGGCT
Smutans_sanger_rev_revcomp  CTGGGGATTACCTCAAAGCTGCTAAGGGGATCCATAAAAATGATAAGGCT
                            **************************************************

Smutans_amplicon            GCTAATGATCATTTGTCTATTTTAGAGGCATGGAGTGACAAC-GACACTC
Smutans_sanger_rev_revcomp  GCTAATGATCATTTGTCTATTTTAGAGGCATGGAGTTATAACTGATACTC
                            ************************************ * *** ** ****

Smutans_amplicon            CTTACCTTCATGATGATGGCGACAATATGATTAATATGGACAATAAGCTG
Smutans_sanger_rev_revcomp  CTTACCTTCATGATGATGGCGACAATATGATTAATATGGACAATAGGCTA
                            ********************************************* *** 

Smutans_amplicon            CGTTTGTCTCTATTATTTTCATTAGCTAAACCCTTAAATCAACGTTCAGG
Smutans_sanger_rev_revcomp  CGTCT-TCTCT------------------------------GCT-----T
                            *** * *****                              *        

Smutans_amplicon            CA
Smutans_sanger_rev_revcomp  TC
```

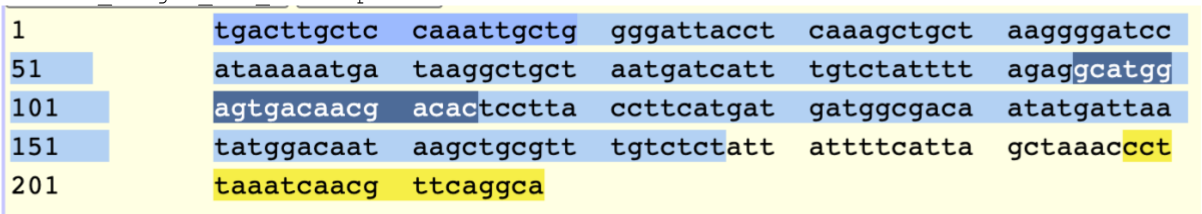

Figure 21. MSA for S. mutans Sanger reverse complement for the reverse reading sequencing results superimposed on Primer3Plus output of amplicon.

The entire target amplicon sequence for S. mutans is shown, with forward primer (purple), probe (black), reverse primer (yellow) and multiple sequence alignment result for the amplicon and the Sanger sequencing product (blue highlight)

Table 11. Multiple sequence alignment results for S. wiggsiae- target amplicon and Sanger sequencing Forward reading[a]

```
CLUSTAL W (1.83) multiple sequence alignment

Swiggsiae_amplicon    TGGTGAGTGGACTTTATGAATAAGCACCGGCTAACTACGTGCCAGCAGCC
Swiggsiae_sanger_fwd  CA----------------------------------CGTG-CAGCAGCC
                                                          **** ********

Swiggsiae_amplicon    GCGGTAATACGTAGGGTGCAAGCGTTGTCCGGATTTATTGGGCGTAAAGG
Swiggsiae_sanger_fwd  GCGGT-ATACGTAGGGTGCAAGCGTTGTCCGGATTTATTGGGCGTAAAGG
                      ***** ********************************************

Swiggsiae_amplicon    GCTCGTAGGCGGTTTGTTGCGTCTGGTGTGAAAGCTTACTGCTTAACGGT
Swiggsiae_sanger_fwd  GCTCGTAGGCGGTTTGTTGCGTCTGGTGTGAAAGCTTACTGCTTAACGGT
                      **************************************************

Swiggsiae_amplicon    AGGTTGCGCT-G
Swiggsiae_sanger_fwd  AGGTTGCGCTGA
```

```
**********
```

Legend: Blue highlight denotes an aligned sequence, with exception of single nucleotide polymorphism (shown in yellow highlight)

[a] The first 125 base pairs from the Sanger sequencing forward reading were used in the multiple sequencing alignment as per the output from the chromatogram in Figure 22.

1	TGGTGAGTGG	ACTTTATGAA	TAAGCACCGG	CTAACTACGT	GCCAGCAGCC
51	GCGGTAATAC	GTAGGGTGCA	AGCGTTGTCC	GGATTTATTG	GGCGTAAAGG
101	GCTCGTAGGC	GGTTTGTTGC	GTCTGGTGTG	AAAGCTTACT	GCTTAACGGT
151	AGGTTGCGCT	G			

Figure 23. MSA for S. wiggsiae Sanger forward reading sequencing results (including SNPs) superimposed on Primer3Plus output of amplicon.

The entire target amplicon sequence for C durum is shown, with forward primer (purple), probe (black), reverse primer (yellow) and multiple sequence alignment result for the amplicon and the Sanger sequencing product (blue highlight)

Table 12. Multiple sequence alignment results for S. wiggsiae target amplicon and Sanger sequencing reverse complement of the reverse reading [a]

```
CLUSTAL W (1.83) multiple sequence alignment

Swiggsiae_amplicon            TG-GTGAGTGGACTTTATGAATAAGCACCGGCTAACTACGTGCCAGCAGC
Swiggsiae_sanger_rev_revcomp  TTGGTGAGTGGACTTTATGAATAAGCACCGGCTAACTACGTGCCAGCAGC
                              *  ***********************************************

Swiggsiae_amplicon            CGCGGTAATACGTAGGGTGCAAGCGTTGTCCGGATTTATTGGGCGTAAAG
Swiggsiae_sanger_rev_revcomp  CGCGGTAATACGTAGGGTGCAAGCGTTGTCCGGATTTATTGGGCGTAAAG
                              **************************************************

Swiggsiae_amplicon            GGCTCGTAGGCGGTTTGTTGCGTCTGGTGTGAAAGCTTACTGCTTAACGG
Swiggsiae_sanger_rev_revcomp  GGCTCGTAGA-----------------------------------------
                              *********

Swiggsiae_amplicon            TAGGTTGCGCTG
Swiggsiae_sanger_rev_revcomp  ----------CG
```

Legend: Blue highlight denotes an aligned sequence, with exception of single nucleotide polymorphism (shown in yellow highlight)

[a] Sanger sequencing segment used from base pair 289 to 401 from chromatogram output in Figure 24.

1	TGGTGAGTGG	ACTTTATGAA	TAAGCACCGG	CTAACTACGT	GCCAGCAGCC
51	GCGGTAATAC	GTAGGGTGCA	AGCGTTGTCC	GGATTTATTG	GGCGTAAAGG
101	GCTCGTAGGC	GGTTTGTTGC	GTCTGGTGTG	AAAGCTTACT	GCTTAACGGT
151	AGGTTGCGCT	G			

Figure 25. MSA for S. wiggsiae Sanger reverse complement for the reverse reading sequencing results superimposed on Primer3Plus output of amplicon.

The entire target amplicon sequence for C durum is shown, with forward primer (purple), probe (black), reverse primer (yellow) and multiple sequence alignment result for the amplicon and the Sanger sequencing product (blue highlight)
